# Supplementary material for: Study on the Dynamic Changes in Non-Volatile Metabolites of Rizhao Green Tea Based on Metabolomics
Source: Molecules. 2023 Nov 6;28(21):7447. doi: 10.3390/molecules28217447 (PMC10650644; doi:10.3390/molecules28217447)
Supplement: Supplementary file 1 [file molecules-28-07447-s001.zip › molecules-2624124-supplementary.pdf]

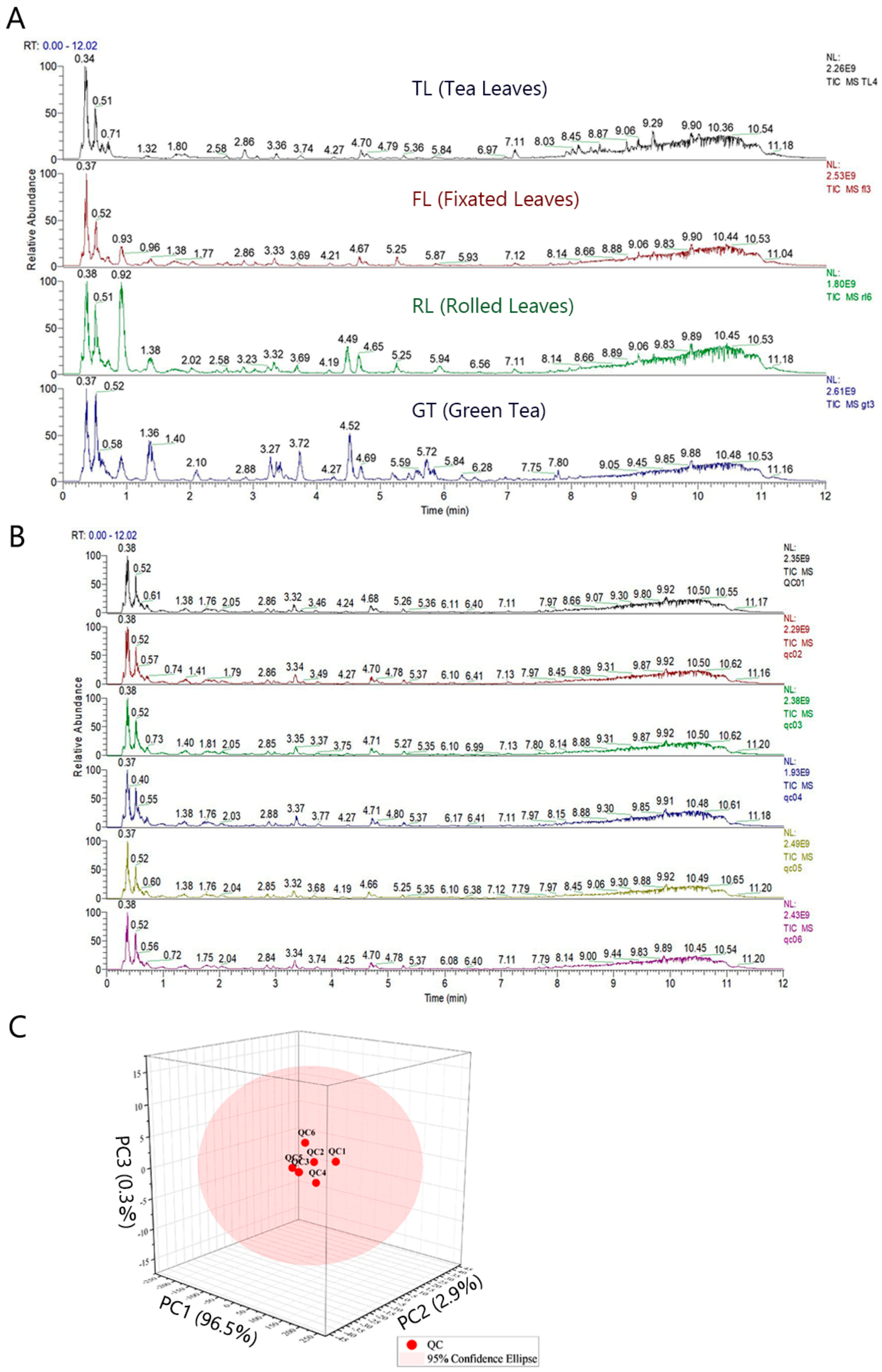

Figure S1. Obtained by C18 column in ESI− mode (A) TIC of tea samples with different processing (B) TIC of QC samples (C) 3D-principal component analysis of samples.

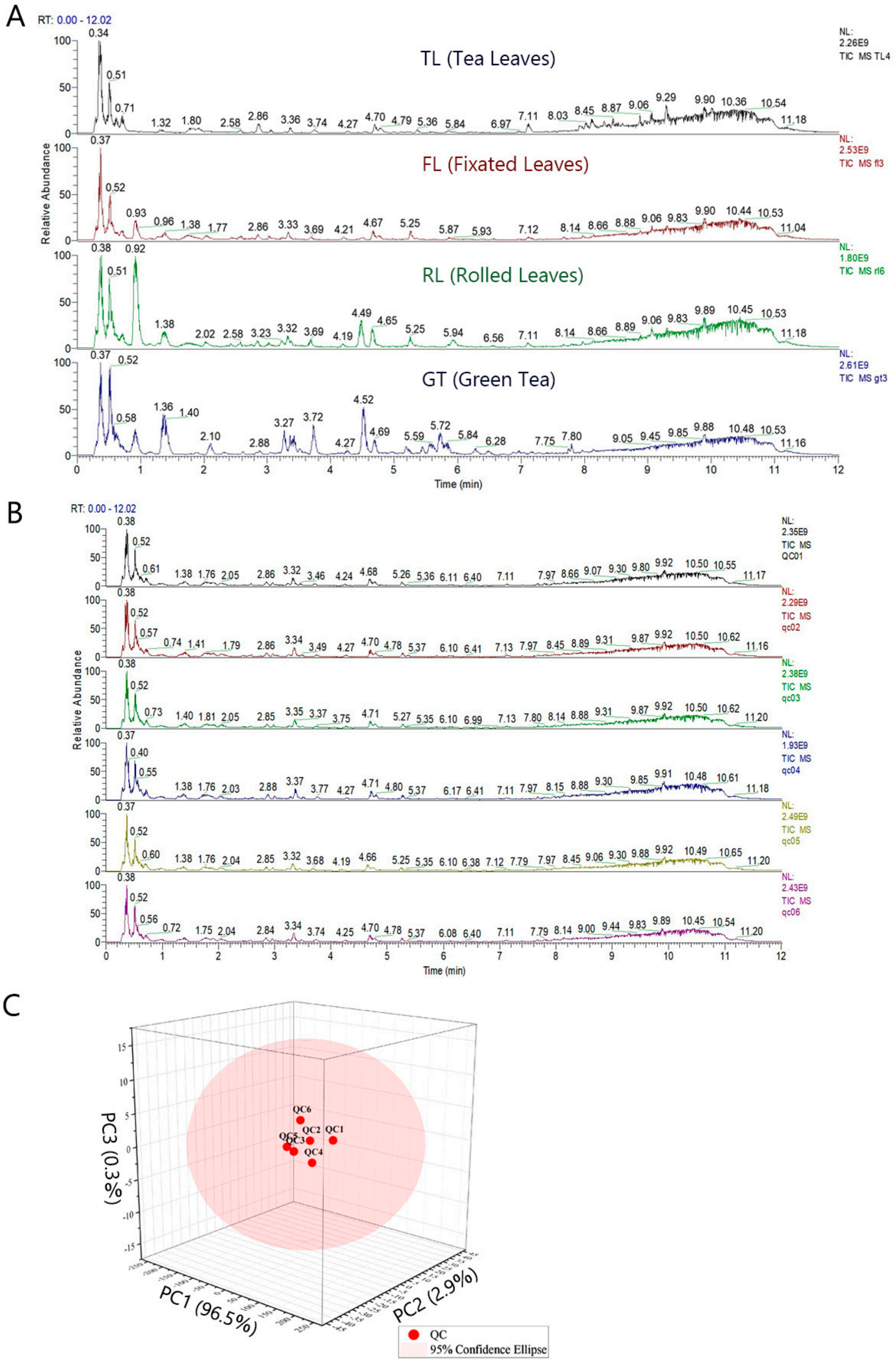

Figure S2. Obtained by Hilic column in ESI+ mode (A) TIC of tea samples with different processing (B) TIC of QC samples (C) 3D-principal component analysis of samples.

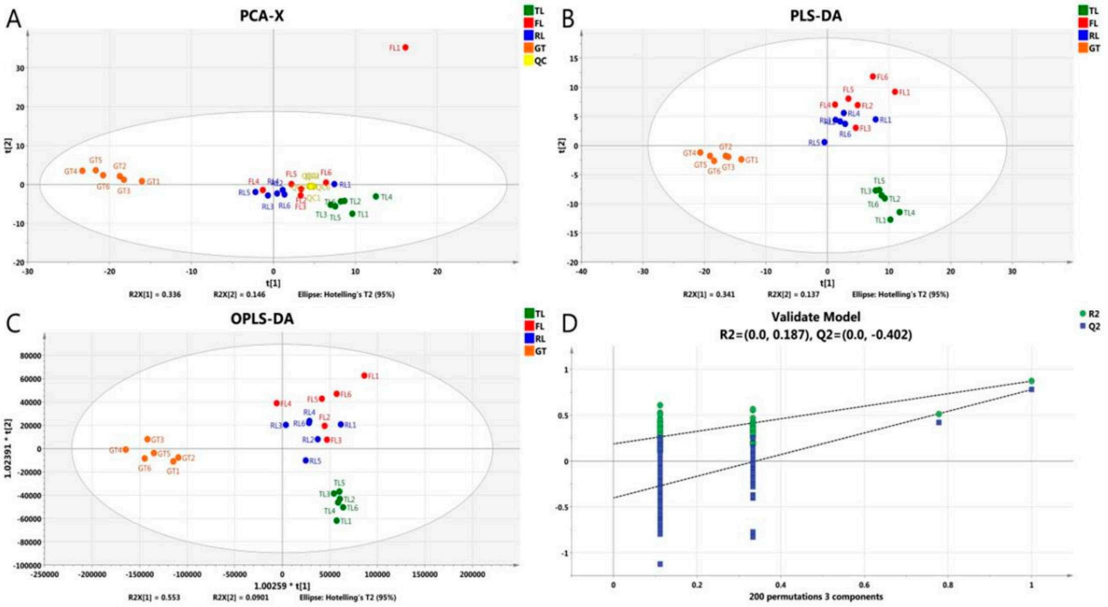

Figure S3. Multivariate statistical analyses of Rizhao green tea samples at different processing stages (C18 column, ESI−.).

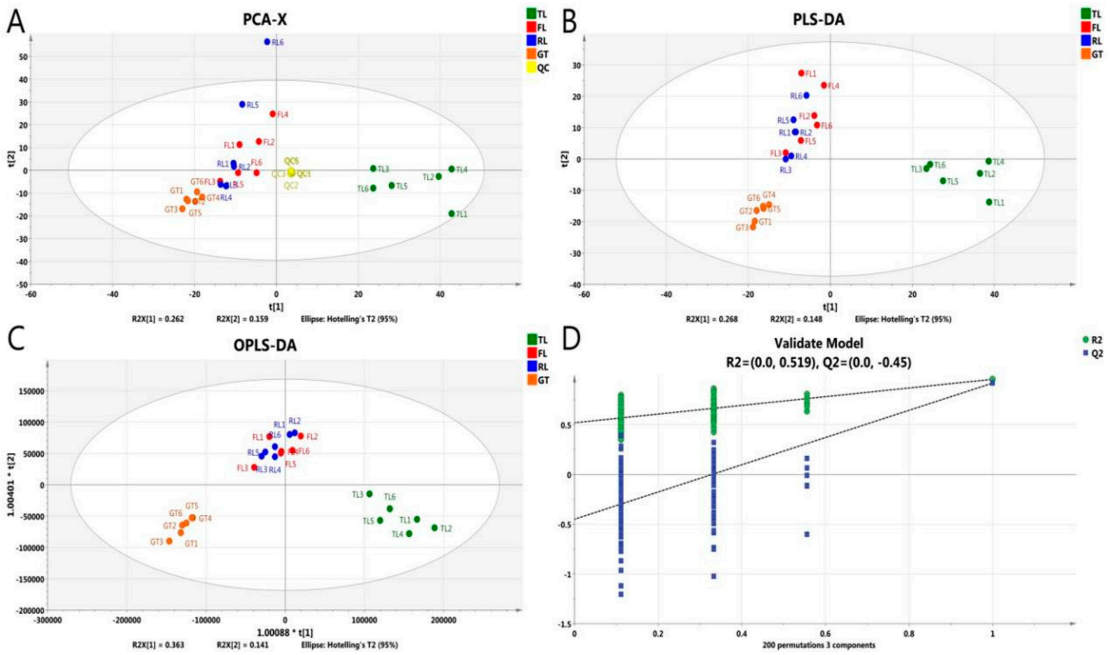

Figure S4. Multivariate statistical analyses of Rizhao green tea samples at different processing stages (Hilic column, ESI+).

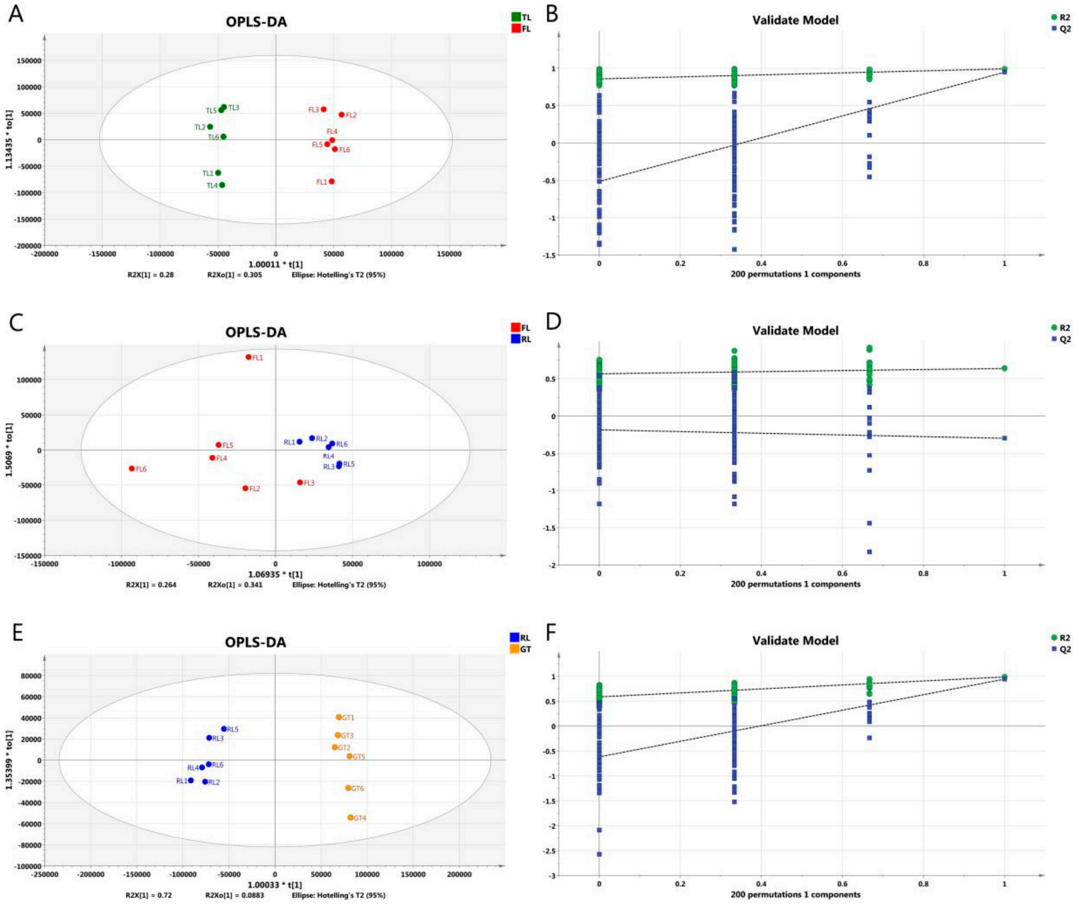

Figure S5. OPLS-DA analysis and permutation tests in samples at adjacent processing stages.

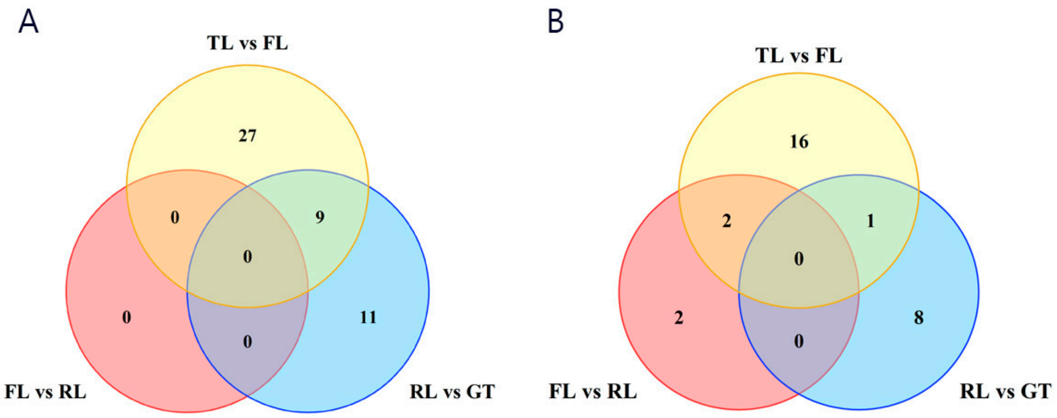

Figure S6. Venn diagram of differential metabolites of (A) C18 column in ESI− mode (B) Hilic column in ESI+ mode.

Table S1. List of 530 non-volatile metabolites in the processing of Rizhao green tea (ESI+, C18 column).

NO	Name	Formula	CAS	Molecular Weight (Da)	Annot.Delta Mass (Da)
1	1-Aminocyclopropanecarboxylic acid	$C_4H_7NO_2$	22059-21-8	101.05	-0.00022
2	L-beta-Homoserine	$C_4H_9NO_3$	16504-56-6	119.06	-0.00014
3	L-Cysteine	$C_3H_7NO_2S$	52-90-4	121.02	-0.00031
4	L-Pyroglutamic acid	$C_5H_7NO_3$	98-79-3	129.04	-0.00013
5	Pipecolic acid	$C_6H_{11}NO_2$	535-75-1	129.08	-0.00023
6	L-Norleucine	$C_6H_{13}NO_2$	327-57-1	131.09	-0.00021
7	N,N-Diethylglycine	$C_6H_{13}NO_2$	1606-01-5	131.09	-0.00021
8	Aminocaproic acid	$C_6H_{13}NO_2$	1319-82-0	131.09	-0.00018
9	Allysine	$C_6H_{11}NO_3$	1962-83-0	145.07	-0.00015
10	Acetylserine	$C_5H_9NO_4$	16354-58-8	147.05	-0.00024
11	3-Methylcrotonylglycine	$C_7H_{11}NO_3$	33008-07-0	157.07	-0.00034
12	L-Phenylalanine	$C_9H_{11}NO_2$	63-91-2	165.08	-0.0002
13	N-Acetyl-L-leucine	$C_8H_{15}NO_3$	1188-21-2	173.11	-0.00007
14	Boc-Glycine	$C_7H_{13}NO_4$	4530-20-5	175.08	-0.00018
15	Ethyl methioninate	$C_7H_{15}NO_2S$	452-95-9	177.08	-0.0003
16	DL-Tyrosine	$C_9H_{11}NO_3$	556-03-6	181.07	-0.00016
17	1-(Ethoxycarbonyl)proline	$C_8H_{13}NO_4$	5700-74-3	187.08	-0.00024
18	N-Glycyl-L-leucine	$C_8H_{16}N_2O_3$	869-19-2	188.12	-0.00051
19	Glycyl-L-norleucine	$C_8H_{16}N_2O_3$	1504-41-2	188.12	-0.00041
20	N-Isopropyl-L-glutamine	$C_8H_{16}N_2O_3$	4311-12-0	188.12	-0.0002
21	N-Acetyl-L-glutamic acid	$C_7H_{11}NO_5$	1188-37-0	189.06	0.00015
22	Dihydroxyphenylalanine	$C_9H_{11}NO_4$	59-92-7	197.07	-0.00022
23	N^2,N^2-Diethylglutamine	$C_9H_{18}N_2O_3$	ChemSpider ID 476314	202.13	-0.00025
24	Alanylleucine	$C_9H_{18}N_2O_3$	1999-42-4	202.13	-0.00019
25	DL-Tryptophan	$C_{11}H_{12}N_2O_2$	54-12-6	204.09	-0.00031
26	Cinnamoylglycine	$C_{11}H_{11}NO_3$	16534-24-0	205.07	-0.00044
27	Val-Pro	$C_{10}H_{18}N_2O_3$	20488-27-1	214.13	-0.00019
28	2-Hydroxyoct-7-enoylglycine	$C_{10}H_{17}NO_4$	ChemSpider ID 74849653	215.12	-0.00033
29	N-(4-Oxobutyl)-L-glutamine	$C_9H_{16}N_2O_4$	ChemSpider ID 26332249	216.11	-0.0002
30	N-Butyl-4-methylglutamine	$C_{10}H_{20}N_2O_3$	ChemSpider ID 492612	216.15	-0.00037
31	Val-Val	$C_{10}H_{20}N_2O_3$	3918-94-3	216.15	-0.00031
32	Boc-5-Aminopentanoic acid	$C_{10}H_{19}NO_4$	27219-07-4	217.13	-0.00036
33	5-Hydroxy-DL-tryptophan	$C_{11}H_{12}N_2O_3$	56-69-9	220.08	-0.00025
34	4-(Acetylamino)phenylalanine	$C_{11}H_{14}N_2O_3$	24250-87-1	222.10	-0.00034
35	Gly-Phe	$C_{11}H_{14}N_2O_3$	721-66-4	222.10	-0.0003

NO	Name	Formula	CAS	Molecular Weight (Da)	Annot.Delta Mass (Da)
36	Leu-pro	$C_{11}H_{20}N_2O_3$	6403-35-6	228.15	-0.00036
37	2,6-Diacetamidohexanoic acid	$C_{10}H_{18}N_2O_4$	35436-74-9	230.13	-0.00034
38	Leu-Val	$C_{11}H_{22}N_2O_3$	35436-83-0	230.16	-0.00044
39	Val-Leu	$C_{11}H_{22}N_2O_3$	22906-55-4	230.16	-0.0004
40	Boc-L-Asparagine	$C_9H_{16}N_2O_5$	7536-55-2	232.11	-0.00051
41	Threonylleucine	$C_{10}H_{20}N_2O_4$	50299-12-2	232.14	-0.00042
42	Leucyl-4-hydroxyproline	$C_{11}H_{20}N_2O_4$	ChemSpider ID 20170035	244.14	-0.00045
43	Leu-Leu	$C_{12}H_{24}N_2O_3$	3303-31-9	244.18	-0.00058
44	Leu-Gly-Gly	$C_{10}H_{19}N_3O_4$	4337-37-5	245.14	-0.00045
45	Boc-7-Aminoheptanoic acid	$C_{12}H_{23}NO_4$	60142-89-4	245.16	-0.00038
46	Boc-Lysine	$C_{11}H_{22}N_2O_4$	13734-28-6	246.16	-0.00046
47	N(2)-Succinyl-L-glutamic acid	$C_9H_{13}NO_7$	33981-72-5	247.07	-0.00039
48	Ala-Tyr	$C_{12}H_{16}N_2O_4$	3061-88-9	252.11	-0.00062
49	Leu-Gln	$C_{11}H_{21}N_3O_4$	ChemSpider ID 3511125	259.15	-0.00062
50	Lys-Leu	$C_{12}H_{25}N_3O_3$	7369-79-1	259.19	-0.00061
51	alpha-Glutamyl-4-hydroxyproline	$C_{10}H_{16}N_2O_6$	ChemSpider ID 35032069	260.10	-0.00042
52	L-gamma-Glutamyl-L-leucine	$C_{11}H_{20}N_2O_5$	2566-39-4	260.14	-0.00052
53	Leu-Glu	$C_{11}H_{20}N_2O_5$	16364-31-1	260.14	-0.00051
54	Phenylac-gln-OH	$C_{13}H_{16}N_2O_4$	28047-15-6	264.11	-0.0006
55	N-Valylphenylalanine	$C_{14}H_{20}N_2O_3$	75946-40-6	264.15	-0.00061
56	(2S,4S)-Hypoglycin B	$C_{12}H_{18}N_2O_5$	ChemSpider ID 390178	270.12	-0.00067
57	gamma-L-Glutamyl-L-glutamic acid	$C_{10}H_{16}N_2O_7$	1116-22-9	276.10	-0.00058
58	Leu-Phe	$C_{15}H_{22}N_2O_3$	56217-82-4	278.16	-0.00068
59	alpha-Asp-Phe	$C_{13}H_{16}N_2O_5$	13433-09-5	280.11	-0.00074
60	N6-((Benzyloxy)carbonyl)lysine	$C_{14}H_{20}N_2O_4$	32302-83-3	280.14	-0.00059
61	Indoleacetylaspartate	$C_{14}H_{14}N_2O_5$	2456-73-7	290.09	-0.0008
62	N-(2-Amino-3-phenylpropanoyl)glutamine	$C_{14}H_{19}N_3O_4$	ChemSpider ID 35032821	293.14	-0.00058
63	Aspartame	$C_{14}H_{18}N_2O_5$	22839-47-0	294.12	-0.00067
64	Leucyltyrosine	$C_{15}H_{22}N_2O_4$	968-21-8	294.16	-0.00077
65	Ile-Tyr	$c_{15}H_{22}N_2O_4$	38579-21-4	294.16	-0.00068
66	Nicotianamine	$C_{12}H_{21}N_3O_6$	34441-14-0	303.14	-0.00044
67	Tyr-Gln	$C_{14}H_{19}N_3O_5$	28252-40-6	309.13	-0.00083
68	N-(tert-Butoxycarbonyl)-4-nitro-L-phenylalanine	$C_{14}H_{18}N_2O_6$	33305-77-0	310.12	-0.00074
69	S-Methylglutathione	$C_{11}H_{19}N_3O_6S$	2922-56-7	321.10	-0.00077

NO	Name	Formula	CAS	Molecular Weight (Da)	Annot.Delta Mass (Da)
70	(Ac)2-L-Lysyl-D-alanyl-D-alanine	$C_{16}H_{28}N_4O_6$	24570-39-6	372.20	-0.00092
71	Oxidized glutathione	$C_{20}H_{32}N_6O_{12}S_2$	121-24-4	612.15	-0.00101
72	Liquiritigenin	$C_{15}H_{12}O_4$	578-86-9	256.07	-0.00064
73	Genistein	$C_{15}H_{10}O_5$	446-72-0	270.05	-0.00073
74	Naringenin chalcone	$C_{15}H_{12}O_5$	73692-50-9	272.07	-0.00082
75	Naringenin	$C_{15}H_{12}O_5$	480-41-1	272.07	-0.00068
76	Kaempferol	$C_{15}H_{10}O_6$	520-18-3	286.05	-0.00076
77	Luteolin	$C_{15}H_{10}O_6$	491-70-3	286.05	-0.00072
78	Aromadendrin	$C_{15}H_{12}O_6$	480-20-6	288.06	-0.00073
79	Tetrahydroxyflavanone	$C_{15}H_{12}O_6$	20725-03-5	288.06	-0.00071
80	Cianidanol	$C_{15}H_{14}O_6$	154-23-4	290.08	-0.00077
81	Quercetin	$C_{15}H_{10}O_7$	117-39-5	302.04	-0.0008
82	3,5,7,2’,4’-Pentahydroxyflavone	$C_{15}H_{10}O_7$	480-16-0	302.04	-0.00079
83	2’,3,5,6’,7-Pentahydroxyflavanone	$C_{15}H_{12}O_7$	80366-15-0	304.06	-0.00075
84	Myricetin	$C_{15}H_{10}O_8$	529-44-2	318.04	-0.00072
85	Flavan-3,3’,4,4’,5,5’,7-heptol	$C_{15}H_{14}O_8$	491-52-1	322.07	-0.00092
86	6-Methoxyquercetin	$C_{16}H_{12}O_8$	519-96-0	332.05	-0.00078
87	Theogallin	$C_{14}H_{16}O_{10}$	17365-11-6	344.07	-0.00076
88	Epiafzelechin 3-O-gallate	$C_{22}H_{18}O_9$	108907-43-3	426.09	-0.00101
89	Isovitexin	$C_{21}H_{20}O_{10}$	29702-25-8	432.10	-0.00106
90	Vitexin	$C_{21}H_{20}O_{10}$	3681-93-4	432.10	-0.00096
91	Swertianolin	$C_{20}H_{20}O_{11}$	23445-00-3	436.10	-0.00081
92	ECG	$C_{22}H_{18}O_{10}$	1257-08-5	442.09	-0.00129
93	Trifolin	$C_{21}H_{20}O_{11}$	23627-87-4	448.10	-0.00117
94	EGCG	$C_{22}H_{18}O_{11}$	989-51-5	458.08	-0.00113
95	Quercetin 4’-O-glucoside	$C_{21}H_{20}O_{12}$	20229-56-5	464.09	-0.00107
96	Quercetin-3β-D-glucoside	$C_{21}H_{20}O_{12}$	482-35-9	464.09	-0.00106
97	Silandrin	$C_{25}H_{22}O_9$	70815-32-6	466.13	-0.00126
98	Myricetin 3-galactoside	$C_{21}H_{20}O_{13}$	15648-86-9	480.09	-0.00099
99	Corymboside	$C_{26}H_{28}O_{14}$	73543-87-0	564.15	-0.00118
100	Apigenin-8-C-glucoside	$C_{27}H_{30}O_{14}$	64820-99-1	578.16	-0.00148
101	Tiliroside	$C_{30}H_{26}O_{13}$	20316-62-5	594.14	-0.00106
102	Kaempferol 3-O-rutinoside	$C_{27}H_{30}O_{15}$	17650-84-9	594.16	-0.00145
103	Rutin	$C_{27}H_{30}O_{16}$	153-18-4	610.15	-0.00144
104	Quercetin 3-O-Rhamnoside-7-O-glucoside	$C_{27}H_{30}O_{16}$	17306-45-5	610.15	-0.00137
105	Mauritianin	$C_{33}H_{40}O_{19}$	109008-28-8	740.21	-0.0023
106	3,4-Dihydroxybenzaldehyde	$C_7H_6O_3$	139-85-5	138.03	-0.00032
107	4-Hydroxybenzoic acid	$C_7H_6O_3$	99-96-7	138.03	-0.00027
108	Salicylic acid	$C_7H_6O_3$	69-72-7	138.03	-0.00024
109	Protocatechuic acid	$C_7H_6O_4$	99-50-3	154.03	-0.00026

NO	Name	Formula	CAS	Molecular Weight (Da)	Annot.Delta Mass (Da)
110	o-Coumaric acid	$C_9H_8O_3$	614-60-8	164.05	-0.00035
111	4-Hydroxycinnamic acid	$C_9H_8O_3$	501-98-4	164.05	-0.0003
112	4-Hydroxyphenylglyoxylic acid	$C_8H_6O_4$	15573-67-8	166.03	-0.0002
113	3,4-Dihydroxyphenylacetic acid	$C_8H_8O_4$	102-32-9	168.04	-0.00029
114	Gallic acid	$C_7H_6O_5$	149-91-7	170.02	-0.00019
115	Shikimic acid	$C_7H_{10}O_5$	138-59-0	174.05	-0.00037
116	Caffeoquinone	$C_9H_6O_4$	15416-77-0	178.03	-0.00038
117	4-Methoxycinnamic acid	$C_{10}H_{10}O_3$	830-09-1	178.06	-0.00019
118	Caffeic acid	$C_9H_8O_4$	331-39-5	180.04	-0.00025
119	2,5-Dihydroxyterephthalic acid	$C_8H_6O_6$	610-92-4	198.02	-0.00031
120	Syringic acid	$C_9H_{10}O_5$	530-57-4	198.05	-0.00024
121	3-Acetoxycinnamic acid	$C_{11}H_{10}O_4$	20375-42-2	206.06	-0.00035
122	Methyl 3-(2,3-dihydroxy-3-methylbutyl)-4-hydroxybenzoate	$C_{13}H_{18}O_5$	117176-70-2	254.11	-0.0005
123	(2R,3S)-trans-Coutaric acid	$C_{13}H_{12}O_8$	27174-06-7	296.05	-0.00075
124	Caftaric acid	$C_{13}H_{12}O_9$	67879-58-7	312.05	-0.00067
125	Digallic acid	$C_{14}H_{10}O_9$	536-08-3	322.03	-0.00077
126	Coniferyl ferulate	$C_{20}H_{20}O_6$	63644-62-2	356.13	-0.00088
127	2-(2-Carboxyethyl)-5-hydroxyphenyl hexopyranosiduronic acid	$C_{15}H_{18}O_{10}$	ChemSpider ID 74886628	358.09	-0.00086
128	Sorbic acid	$C_6H_8O_2$	110-44-1	112.05	-0.00016
129	7-Octene-3,5-diyn-1-ol	C_8H_8O	114020-45-0	120.06	-0.00025
130	9-Oxodecenoic acid	$C_{10}H_{16}O_3$	334-20-3	184.11	-0.00027
131	12-Aminolauric Acid	$C_{12}H_{25}NO_2$	693-57-2	215.19	-0.00039
132	12-Hydroxyjasmonic acid	$C_{12}H_{18}O_4$	140631-27-2	226.12	-0.00052
133	Dodecylethanol amide	$C_{14}H_{29}NO_2$	142-78-9	243.22	-0.00044
134	Hexadecanamide	$C_{16}H_{33}NO$	629-54-9	255.26	-0.00065
135	Phloretin	$C_{15}H_{14}O_5$	60-82-2	274.08	-0.00078
136	α-Linolenic acid	$C_{18}H_{30}O_2$	463-40-1	278.22	-0.00063
137	Linolenelaidic acid	$C_{18}H_{30}O_2$	28290-79-1	278.22	-0.00056
138	12-oxo Phytodienoic acid	$C_{18}H_{28}O_3$	85551-10-6	292.20	-0.00079
139	Methyl alpha-eleostearate	$C_{19}H_{32}O_2$	4175-47-7	292.24	-0.00076
140	6-Tuliposide B	$C_{11}H_{18}O_9$	244105-18-8	294.09	-0.00053
141	Tuliposide B	$C_{11}H_{18}O_9$	19870-33-8	294.09	-0.00052
142	6-Gingerol	$C_{17}H_{26}O_4$	23513-14-6	294.18	-0.00063
143	9-oxo-10E,12Z-Octadecadienoic acid	$C_{18}H_{30}O_3$	54232-59-6	294.22	-0.0007
144	9-oxo-10,12-Octadecadienoic acid	$C_{18}H_{30}O_3$	54232-58-5	294.22	-0.00069
145	Ricinelaidic acid	$C_{18}H_{34}O_3$	141-22-0	298.25	-0.00075
146	Palmitoyl ethanolamide	$C_{18}H_{37}NO_2$	544-31-0	299.28	-0.00066
147	9-J1-Phytoprostane	$C_{18}H_{28}O_4$	ChemSpider ID	308.20	-0.0008

NO	Name	Formula	CAS	Molecular Weight (Da)	Annot.Delta Mass (Da)
			32033755		
148	8-Gingerol	$C_{19}H_{30}O_4$	23513-08-8	322.21	-0.00089
149	Linoleoyl ethanolamide	$C_{20}H_{37}NO_2$	68171-52-8	323.28	-0.0007
150	Oleoyl ethanolamide	$C_{20}H_{39}NO_2$	111-58-0	325.30	-0.00071
151	10-Nitrooleic acid	$C_{18}H_{33}NO_4$	875685-46-4	327.24	-0.00087
152	9-Nitro-9E-octadecenoic acid	$C_{18}H_{33}NO_4$	875685-44-2	327.24	-0.00074
153	Glyceryl palmitate	$C_{19}H_{38}O_4$	542-44-9	330.28	-0.00084
154	9,10,13-Trihydroxyoctadecanoic acid	$C_{18}H_{36}O_5$	ChemSpider ID 24022689	332.26	-0.00107
155	3-oxo-N-[(3S)-2-Oxotetrahydro-3-furanyl]hexadecanamide	$C_{20}H_{35}NO_4$	925448-37-9	353.26	-0.00119
156	N-Oleoyl Alanine	$C_{21}H_{39}NO_3$	745733-78-2	353.29	-0.00098
157	(5Z,13E,15R)-15-Hydroxy-16,16-dimethyl-9-oxoprosta-5,13-dien-1-oic acid	$C_{22}H_{36}O_4$	53658-98-3	364.26	-0.00066
158	N-Oleoyl-L-Serine	$C_{21}H_{39}NO_4$	1246302-99-7	369.29	-0.001
159	Brassicasterol	$C_{28}H_{42}O$	516-85-8	394.32	-0.00086
160	Cholesteryl acetate	$C_{29}H_{48}O_2$	604-35-3	428.36	-0.001
161	Halosterol B	$C_{29}H_{48}O_2$	ChemSpider ID 9808066	428.36	-0.0007
162	1-Hexadecanoyl-sn-glycero-3-phosphoethanolamine	$C_{21}H_{44}NO_7P$	53862-35-4	453.28	-0.001
163	1-Palmitoylphosphatidylcholine	$C_{24}H_{50}NO_7P$	17364-18-0	495.33	-0.00119
164	Soyacerebroside I	$C_{40}H_{75}NO_9$	114297-20-0	713.54	-0.00178
165	L-Dilinolenoyl lecithin	$C_{44}H_{76}NO_8P$	2701-19-1	777.53	-0.00168
166	Cytosine	$C_4H_5N_3O$	71-30-7	111.04	-0.00017
167	5-Formamidopyrimidine	$C_5H_5N_3O$	56621-84-2	123.04	-0.00018
168	5-Methylcytosine	$C_5H_7N_3O$	554-01-8	125.06	-0.0001
169	Thymine	$C_5H_6N_2O_2$	65-71-4	126.04	-0.00028
170	Adenine	$C_5H_5N_5$	73-24-5	135.05	-0.00029
171	Hypoxanthine	$C_5H_4N_4O$	68-94-0	136.04	-0.00016
172	Guanine	$C_5H_5N_5O$	73-40-5	151.05	-0.00018
173	2,6-Diamino-8-azapurine	$C_4H_5N_7$	18620-97-8	151.06	0.00016
174	Xanthine	$C_5H_4N_4O_2$	69-89-6	152.03	-0.00024
175	1-Methylguanine	$C_6H_7N_5O$	938-85-2	165.06	-0.0002
176	6-O-Methylguanine	$C_6H_7N_5O$	20535-83-5	165.06	-0.00013
177	1-Methylxanthine	$C_6H_6N_4O_2$	6136-37-4	166.05	-0.00027
178	7-Methylxanthine	$C_6H_6N_4O_2$	552-62-5	166.05	-0.00026
179	trans-Zeatin	$C_{10}H_{13}N_5O$	1637-39-4	219.11	-0.00028
180	5-Methyldeoxycytidine	$C_{10}H_{15}N_3O_4$	838-07-3	241.11	-0.00035
181	1-Pentofuranosyldihydro-2,4(1H,3H)-pyrimidinedione	$C_9H_{14}N_2O_6$	30100-83-5	246.08	-0.00049

NO	Name	Formula	CAS	Molecular Weight (Da)	Annot.Delta Mass (Da)
182	2’-Deoxyadenosine	$C_{10}H_{13}N_5O_3$	958-09-8	251.10	-0.00049
183	1-(beta-D-Ribofuranosyl)-1,4-dihydronicotinamide	$C_{11}H_{16}N_2O_5$	19132-12-8	256.11	-0.00062
184	Adenosine	$C_{10}H_{13}N_5O_4$	58-61-7	267.10	-0.00051
185	5’-Amino-5’-oxo-5’-deoxyadenosine	$C_{10}H_{12}N_6O_4$	35788-21-7	280.09	-0.00059
186	1-Methyladenosine	$C_{11}H_{15}N_5O_4$	15763-06-1	281.11	-0.00054
187	2’-O-Methyladenosine	$C_{11}H_{15}N_5O_4$	2140-79-6	281.11	-0.00053
188	Guanosine	$C_{10}H_{13}N_5O_5$	118-00-3	283.09	-0.00059
189	N6,N6-Dimethyladenosine	$C_{12}H_{17}N_5O_4$	2620-62-4	295.13	-0.00047
190	5’-S-Methyl-5’-thioadenosine	$C_{11}H_{15}N_5O_3S$	2457-80-9	297.09	-0.00063
191	7-Methylguanosine	$C_{11}H_{15}N_5O_5$	20244-86-4	297.11	-0.0007
192	N2,N2-Dimethylguanosine	$C_{12}H_{17}N_5O5$	2140-67-2	311.12	-0.00067
193	Adenosine-3’-5’-cyclic monophosphate	$C_{10}H_{12}N_5O6P$	86594-35-6	329.05	-0.00058
194	Hypoxanthine deoxyriboside	$C_{10}H_{13}N_4O_7P$	3393-18-8	332.05	0.00028
195	Cyclic guanosine monophosphate	$C_{10}H_{12}N_5O_7P$	7665-99-8	345.05	-0.00062
196	9-Ribosylzeatin	$C_{15}H_{21}N_5O_5$	15896-46-5	351.15	-0.00066
197	N6-Succinyl adenosine	$C_{14}H_{17}N_5O_8$	4542-23-8	383.11	-0.00104
198	Glutaric anhydride	$C_5H_6O_3$	108-55-4	114.03	-0.0003
199	Levulinic acid	$C_5H_8O_3$	123-76-2	116.05	-0.00009
200	(2-Aminoethyl)phosphonic acid	$C_2H_8NO_3P$	2041-14-7	125.02	-0.00048
201	(2S)-2-Amino-4-hexynoic acid	$C_6H_9NO_2$	29834-76-2	127.06	-0.00019
202	1,2,5,6-Tetrahydropyridine-3-carboxylic acid	$C_6H_9NO_2$	498-96-4	127.06	-0.00016
203	6-Methylnicotinic acid	$C_7H_7NO_2$	3222-47-7	137.05	-0.00015
204	6-oxo-Pipecolinic acid	$C_6H_9NO_3$	3770-22-7	143.06	-0.00001
205	Tetrahydro-5-oxo-2-furanacetic acid	$C_6H_8O_4$	60551-20-4	144.04	-0.00004
206	4-Acetamidobutyric acid	$C_6H_{11}NO_3$	3025-96-5	145.07	-0.0001
207	2-Hydroxyglutaric acid	$C_5H_8O_5$	2889-31-8	148.04	0.00011
208	(2Z)-8-Hydroxy-2-octene-4,6-diynoic acid	$C_8H_6O_3$	65367-65-9	150.03	-0.00026
209	5-Aminosalicylic acid	$C_7H_7NO_3$	89-57-6	153.04	-0.00023
210	2-Hydrazinonicotinic acid	$C_6H_7N_3O_2$	435342-14-6	153.05	-0.00018
211	Dimethyl itaconate	$C_7H_{10}O_4$	617-52-7	158.06	-0.00025
212	Phenylbutyric acid	$C_{10}H_{12}O_2$	1821-12-1	164.08	-0.00028
213	D-Xylonic acid	$C_5H_{10}O_6$	4172-43-4	166.05	0.00016
214	Acetylsalicylic acid	$C_9H_8O_4$	50-78-2	180.04	-0.00023

NO	Name	Formula	CAS	Molecular Weight (Da)	Annot.Delta Mass (Da)
215	5-Hydroxyisophthalic acid	$C_8H_6O_5$	618-83-7	182.02	-0.00024
216	Stipitatic acid	$C_8H_6O_5$	4440-39-5	182.02	-0.00024
217	trans-3-Indoleacrylic acid	$C_{11}H_9NO_2$	29953-71-7	187.06	-0.00029
218	1-(Carboxymethyl)piperidine-4-car boxylic acid	$C_8H_{13}NO_4$	53919-19-0	187.08	-0.00027
219	4-Hydroxyquinoline-2-carboxylic acid	$C_{10}H_7NO_3$	492-27-3	189.04	-0.00032
220	3-(4-Methylbenzoyl)acrylic acid	$C_{11}H_{10}O_3$	20972-36-5	190.06	-0.00025
221	4-(Trifluoromethyl)nicotinic acid	$C_7H_4F_3NO_2$	158063-66-2	191.02	-0.00037
222	5-Hydroxyindole-3-acetic acid	$C_{10}H_9NO_3$	1321-73-9	191.06	-0.00024
223	3-Oxooctahydro-4a(2H)-naphthalen ecarboxylic acid	$C_{11}H_{16}O_3$	91352-51-1	196.11	-0.00033
224	Vanillyl mandelic acid	$C_9H_{10}O_5$	55-10-7	198.05	-0.00028
225	(2E)-3-(3,4-Dimethoxyphenyl)acryl ic acid	$C_{11}H_{12}O_4$	14737-89-4	208.07	-0.00035
226	4-[(Ethoxycarbonyl)amino]benzoic acid	$C_{10}H_{11}NO_4$	5180-75-6	209.07	-0.00041
227	Jasmonic acid	$C_{12}H_{18}O_3$	6894-38-8	210.13	-0.00038
228	2-Hydroxy-6-ketononadienedioic acid	$C_9H_{10}O_6$	ChemSpider ID 4444276	214.05	-0.00039
229	Diphenylphosphinic acid	$C_{12}H_{11}O_2P$	1707-03-5	218.05	-0.00043
230	4,5-Dimethoxy-2-nitrobenzoic acid	$C_9H_9NO_6$	4998-07-6	227.04	-0.00046
231	Dimethyl succinylsuccinate	$C_{10}H_{12}O_6$	6289-46-9	228.06	-0.00034
232	Hopantenic acid	$C_{10}H_{19}NO_5$	18679-90-8	233.13	-0.00056
233	3-Amino-3-(4-isopropoxy-3-metho xyphenyl)propanoic acid	$C_{13}H_{19}NO_4$	554402-59-4	253.13	-0.00049
234	3,4,5-Triethoxybenzoic acid	$C_{13}H_{18}O_5$	6970-19-0	254.11	-0.00065
235	10-Heptadecen-8-ynoic acid	$C_{17}H_{28}O_2$	16714-84-4	264.21	-0.00078
236	4-[2-(4-Amino-4-carboxybutanoyl) hydrazino]benzoic acid	$C_{12}H_{15}N_3O_5$	69644-85-5	281.10	-0.0009
237	N-Succinyl-L-diaminopimelic acid	$C_{11}H_{18}N_2O_7$	26605-36-7	290.11	-0.00055
238	(9Z,11E,13S,15Z)-13-Hydroxyocta deca-9,11,15-trienoic acid	$C_{18}H_{30}O_3$	87984-82-5	294.22	-0.00071
239	2’-Deoxymugineic acid	$C_{12}H_{20}N_2O_7$	74235-24-8	304.13	-0.00042
240	Imazamox	$C_{15}H_{19}N_3O_4$	114311-32-9	305.14	-0.00094
241	(9Z,11E)-13-Hydroperoxyoctadeca-9,11-dienoic acid	$C_{18}H_{32}O_4$	23017-93-8	312.23	-0.00092
242	Salicylacyl glucuronide	$C_{13}H_{14}O_9$	29315-53-5	314.06	-0.00085
243	Luteic acid	$C_{14}H_8O_9$	476-67-5	320.02	-0.00059
244	Ethylenediamine-N,N’-diacetic-N,N’-dipropionic acid	$C_{12}H_{20}N_2O_8$	32701-19-2	320.12	-0.00057
245	(2R,9Z,12E,15Z)-2-Hydroperoxy-1	$C_{18}H_{30}O_5$	ChemSpider ID	326.21	-0.00071

NO	Name	Formula	CAS	Molecular Weight (Da)	Annot.Delta Mass (Da)
	3-hydroxy-9,12,15-octadecatrienoic acid		17220740		
246	(2R)-2-[(7Z)-7-Hexadecen-1-yl]-3-methylenesuccinic acid	$C_{21}H_{36}O_4$	ChemSpider ID 29328054	352.26	-0.00078
247	Oxindole	C_8H_7NO	59-48-3	133.05	-0.00017
248	Stachydrine	$C_7H_{13}NO_2$	ChemSpider ID 103115	143.09	-0.0001
249	Daechualkaloid A	$C_{10}H_{13}NO_2$	80933-73-9	179.09	-0.00019
250	Theobromine	$C_7H_8N_4O_2$	83-67-0	180.06	-0.00036
251	Theophylline	$C_7H_8N_4O_2$	58-55-9	180.06	-0.00033
252	Caffeine	$C_8H_{10}N_4O_2$	58-08-2	194.08	-0.00035
253	6-Thiocaffeine	$C_8H_{10}N_4OS$	13182-58-6	210.06	-0.00029
254	N,N-Dimethyl-5-methoxytryptamine	$C_{13}H_{18}N_2O$	1019-45-0	218.14	-0.00057
255	Eseroline	$C_{13}H_{18}N_2O$	469-22-7	218.14	-0.00045
256	Gentisein	$C_{13}H_8O_5$	529-49-7	244.04	-0.00057
257	Echinatine N-oxide	$C_{15}H_{25}NO_6$	20267-93-0	315.17	-0.00079
258	Renardine	$C_{19}H_{27}NO_6$	2318-18-5	365.18	-0.00072
259	Jervine	$C_{27}H_{39}NO_3$	469-59-0	425.29	-0.00105
260	Methyl gallate	$C_8H_8O_5$	99-24-1	184.04	-0.00028
261	Ellagic acid	$C_{14}H_6O_8$	476-66-4	302.01	-0.00075
262	Procyanidin A2	$C_{30}H_{24}O_{12}$	41743-41-3	576.13	-0.00136
263	Procyanidin B2	$C_{30}H_{26}O_{12}$	29106-49-8	578.14	-0.00112
264	Galactosamine	$C_6H_{13}NO_5$	7535-00-4	179.08	-0.00019
265	2-Acetamido-2,6-dideoxyglucose	$C_8H_{15}NO_5$	40614-71-9	205.09	-0.00012
266	2,4-Diacetamido-2,4,6-trideoxy-alpha-D-mannopyranose	$C_{10}H_{18}N_2O_5$	ChemSpider ID 28533246	246.12	-0.00035
267	Methyl 4,6-dideoxy-4-[(3-hydroxypropanoyl)amino]hexopyranoside	$C_{10}H_{19}NO_6$	ChemSpider ID 4928260	249.12	-0.00034
268	1-O-Benzoylpentopyranose	$C_{12}H_{14}O_6$	ChemSpider ID 495245	254.08	-0.00062
269	(2S)-2-(beta-D-Glucopyranosyloxy)-3-methyl-3-butenenitrile	$C_{11}H_{17}NO_6$	66871-89-4	259.11	-0.00046
270	2(R)-Hydroxy-2-methylbutyronitrile-beta-D-glucopyranoside	$C_{11}H_{19}NO_6$	534-67-8	261.12	-0.00041
271	1-Deoxy-1-(hexylamino)hexitol	$C_{12}H_{27}NO_5$	ChemSpider ID 499954	265.19	-0.0004
272	(1S,5R)-5-Isopropenyl-2-methyl-2-cyclohexen-1-yl beta-D-glucopyranoside	$C_{16}H_{26}O_6$	113349-30-7	314.17	-0.00056
273	Methyl	$C_{17}H_{24}N_2O_8$	ChemSpider ID	384.15	-0.00118

NO	Name	Formula	CAS	Molecular Weight (Da)	Annot.Delta Mass (Da)
	2-[({[(benzyloxy)carbonyl]amino}acetyl)amino]-2-deoxyhexopyranoside		511388		
274	6-Acetamido-1,2,3,4-tetra-O-acetyl-6-deoxyhexopyranose	$C_{16}H_{23}NO_{10}$	ChemSpider ID 510192	389.13	-0.00082
275	(2S)-2-{[6-O-(alpha-L-Arabinopyranosyl)-beta-D-glucopyranosyl]oxy}-3-methyl-3-butenenitrile	$C_{16}H_{25}NO_{10}$	79197-21-0	391.15	-0.00083
276	4-O-(2-Acetamido-2-deoxy-beta-D-glucopyranosyl)-beta-D-glucopyranuronic acid	$C_{14}H_{23}NO_{12}$	139626-60-1	397.12	-0.00098
277	1,3,4,6-Tetra-O-acetyl-2-deoxy-2-(1-pyrrolidinyl)hexopyranose	$C_{18}H_{27}NO_9$	ChemSpider ID 499689	401.17	-0.00108
278	Methyl 2-acetamido-2-deoxy-3,6-di-O-methyl-4-O-(2,3,4,6-tetra-O-methyl-beta-D-mannopyranosyl)-beta-D-glucopyranoside	$C_{21}H_{39}NO_{11}$	ChemSpider ID 4933246	481.25	-0.00112
279	Glucosylgalactosyl hydroxylysine	$C_{18}H_{34}N_2O_{13}$	ChemSpider ID 109059	486.21	0.00062
280	2,3,4,6-Tetra-O-acetyl-1-O-[(2E)-3-(3,4-diacetoxyphenyl)-2-propenoyl]hexopyranose	$C_{27}H_{30}O_{15}$	ChemSpider ID 4524029	594.16	-0.00138
281	Pulegone	$C_{10}H_{16}O$	15932-80-6	152.12	-0.00024
282	Zerumbone	$C_{15}H_{22}O$	471-05-6	218.17	-0.00029
283	Caryophyllene oxide	$C_{15}H_{24}O$	1139-30-6	220.18	-0.00041
284	Podolide	$C_{19}H_{22}O_5$	55786-36-2	330.15	-0.00094
285	Gibberellin A7	$C_{19}H_{22}O_5$	510-75-8	330.15	-0.00075
286	Geniposide pentaacetate	$C_{27}H_{34}O_{15}$	49776-64-9	598.19	-0.00173
287	Coumarin	$C_9H_6O_2$	91-64-5	146.04	-0.00033
288	8-Methylcoumarin	$C_{10}H_8O_2$	1807-36-9	160.05	0.00014
289	4-Hydroxycoumarin	$C_9H_6O_3$	1076-38-6	162.03	-0.0003
290	7-Hydroxycoumarine	$C_9H_6O_3$	93-35-6	162.03	-0.0003
291	Hymecromone	$C_{10}H_8O_3$	90-33-5	176.05	-0.00038
292	Esculetin	$C_9H_6O_4$	305-01-1	178.03	-0.00038
293	6-Acetocoumarin	$C_{11}H_8O_3$	1177917-22-4	188.05	-0.00039
294	4,5,7-Trihydroxycoumarin	$C_9H_6O_5$	17575-26-7	194.02	-0.00022
295	Scoparone	$C_{11}H_{10}O_4$	120-08-1	206.06	-0.00034
296	Methoxsalen	$C_{12}H_8O_4$	298-81-7	216.04	-0.00032
297	7-Acetoxy-4-methylcoumarin	$C_{12}H_{10}O_4$	2747-05-9	218.06	-0.00029
298	8-Acetyl-7-hydroxy-4-methylcoumarin	$C_{12}H_{10}O_4$	2555-29-5	218.06	-0.00029

NO	Name	Formula	CAS	Molecular Weight (Da)	Annot.Delta Mass (Da)
299	Urolithin D	$C_{13}H_8O_6$	131086-98-1	260.03	-0.00045
300	(+)-Galbacin	$C_{20}H_{20}O_5$	528-64-3	340.13	-0.00075
301	1,4-Cyclohexadiene	C_6H_8	628-41-1	80.06	-0.00017
302	Cyclohexene	C_6H_{10}	110-83-8	82.08	-0.00026
303	Methyl propiolate	$C_4H_4O_2$	922-67-8	84.02	-0.00019
304	2-Pyrrolidone	C_4H_7NO	616-45-5	85.05	-0.00017
305	4-Penten-1-amine	$C_5H_{11}N$	22537-07-1	85.09	-0.00013
306	Ethyl isothiocyanate	C_3H_5NS	542-85-8	87.01	-0.00018
307	N,N-Dimethylacetamide	C_4H_9NO	127-19-5	87.07	-0.00014
308	1-Heptyne	C_7H_{12}	628-71-7	96.09	-0.0002
309	Cyclopentan-1,3-dion	$C_5H_6O_2$	3859-41-4	98.04	-0.00027
310	Phenylacetylene	C_8H_6	536-74-3	102.05	-0.00015
311	(E)-1-Phenylmethanimine	C_7H_7N	ChemSpider ID 378751	105.06	-0.00011
312	(2E,4E)-2,4-Heptadien-6-ynal	C_7H_6O	7200-04-6	106.04	-0.0002
313	Benzoquinone	$C_6H_4O_2$	106-51-4	108.02	-0.00021
314	alpha-Ocimene	C_8H_{12}	1002-35-3	108.09	-0.00015
315	Hydroquinone	$C_6H_6O_2$	123-31-9	110.04	-0.00022
316	Diacetylene glycol	$C_6H_6O_2$	3031-68-3	110.04	-0.00018
317	3,5-Dimethyl-1,4-hexadiene	C_8H_{14}	761-87-5	110.11	-0.00024
318	1-Vinyl-2-pyrrolidone	C_6H_9NO	88-12-0	111.07	-0.00019
319	N,N-Dimethyl-2-butynamide	C_6H_9NO	53099-32-4	111.07	-0.00015
320	1-Aminopyrrole-2,5-dione	$C_4H_4N_2O_2$	37770-94-8	112.03	-0.00021
321	5-Methyl-3-methylidene-oxolan-2-one	$C_6H_8O_2$	62873-16-9	112.05	-0.0002
322	N-Acetylpyrrolidine	$C_6H_{11}NO$	4030-18-6	113.08	-0.0002
323	N,N-Dimethyl-2-butenamide	$C_6H_{11}NO$	14064-75-6	113.08	-0.00015
324	Butyl isothiocyanate	C_5H_9NS	592-82-5	115.05	-0.00018
325	3-Acetamidopropanal	$C_5H_9NO_2$	73323-68-9	115.06	-0.00018
326	5-(Hydroxymethyl)dihydrofuran-2(3H)-one	$C_5H_8O_3$	10374-51-3	116.05	-0.00017
327	Indole	C_8H_7N	120-72-9	117.06	-0.00014
328	2,4,6-Octatriyn-1-ol	C_8H_6O	13033-21-1	118.04	-0.00011
329	3-Cyanophenol	C_7H_5NO	873-62-1	119.04	-0.00021
330	2-Methyl-5-vinylpyridine	C_8H_9N	140-76-1	119.07	-0.0002
331	4-Hydroxybenzaldehyde	$C_7H_6O_2$	123-08-0	122.04	-0.00033
332	(4Z)-4-Nonen-2-yne	C_9H_{14}	53497-78-2	122.11	-0.00024
333	Hydroxy-1,4-benzoquinone	$C_6H_4O_3$	2474-72-8	124.02	-0.00013
334	Furan-2,5-dicarbaldehyde	$C_6H_4O_3$	823-82-5	124.02	-0.00006
335	1-Acetyl-1,2,3,4-tetrahydropyridine	$C_7H_{11}NO$	19615-27-1	125.08	-0.00025
336	Pyrogallol	$C_6H_6O_3$	87-66-1	126.03	-0.00026
337	5-Hydroxy-6-methyl-2H-pyran-2-o	$C_6H_6O_3$	ChemSpider ID	126.03	-0.00023

NO	Name	Formula	CAS	Molecular Weight (Da)	Annot.Delta Mass (Da)
	ne		4934394		
338	4-Hydroxy-5-methyl-2-methylenefuran-3-one	$C_6H_6O_3$	948557-12-8	126.03	-0.00016
339	5-Methyl-3-vinyl-2-oxazolidinone	$C_6H_9NO_2$	3395-98-0	127.06	-0.00013
340	3-Hydroxy-6-methyl-2,3-dihydro-4H-pyran-4-one	$C_6H_8O_3$	ChemSpider ID 461448	128.05	-0.00024
341	Methyl (2E)-4-oxo-2-pentenoate	$C_6H_8O_3$	2833-24-1	128.05	-0.0002
342	Leucoline	C_9H_7N	91-22-5	129.06	-0.00026
343	Diisopropylethylamine	$C_8H_{19}N$	7087-68-5	129.15	-0.00018
344	Phenylpropynal	C_9H_6O	2579-22-8	130.04	-0.00021
345	(Hydroxyethyl)methacrylate	$C_6H_{10}O_3$	868-77-9	130.06	-0.00014
346	3-Methylindole	C_9H_9N	83-34-1	131.07	-0.00014
347	3-Methylsulfolene	$C_5H_8O_2S$	1193-10-8	132.02	-0.00016
348	Cinnamaldehyde	C_9H_8O	104-55-2	132.06	-0.00025
349	4-Hydroxyindole	C_8H_7NO	2380-94-1	133.05	-0.0002
350	1,9-Decadiyne	$C_{10}H_{14}$	1720-38-3	134.11	-0.0002
351	[1,2,4]Triazolo[1,5-a]pyrimidin-7-ol	$C_5H_4N_4O$	31592-08-2	136.04	-0.00024
352	3-Anisaldehyde	$C_8H_8O_2$	591-31-1	136.05	-0.00025
353	3,4-Dihydroxystyrene	$C_8H_8O_2$	6053-02-7	136.05	-0.00021
354	Phenyl acetate	$C_8H_8O_2$	122-79-2	136.05	-0.00015
355	1-Decen-3-yne	$C_{10}H_{16}$	33622-26-3	136.12	-0.00021
356	1,5,9-Decatriene	$C_{10}H_{16}$	13393-64-1	136.13	-0.00016
357	6-Aminonicotinamide	$C_6H_7N_3O$	329-89-5	137.06	-0.00024
358	(2E)-3-(1H-Imidazol-5-yl)acrylamide	$C_6H_7N_3O$	135200-64-5	137.06	-0.00017
359	Protocatechuic aldehyde	$C_7H_6O_3$	139-85-5	138.03	-0.00027
360	2-Methoxyresorcinol	$C_7H_8O_3$	29267-67-2	140.05	-0.00022
361	Ethyl 2-furoate	$C_7H_8O_3$	1335-40-6	140.05	-0.00015
362	Lepidine	$C_{10}H_9N$	491-35-0	143.07	-0.00008
363	2-Butynophenone	$C_{10}H_8O$	6710-62-9	144.06	-0.00002
364	Indole-4-carboxaldehyde	C_9H_7NO	1074-86-8	145.05	-0.00011
365	N-(4-Hydroxy-1-methylbutyl)acetamide	$C_7H_{15}NO_2$	ChemSpider ID 471526	145.11	-0.00015
366	3,4-Decadiene-6,8-diyn-1-ol	$C_{10}H_{10}O$	6071-59-6	146.07	-0.00007
367	3-(Methylthio)propyl isothiocyanate	$C_5H_9NS_2$	505-79-3	147.02	-0.00032
368	Cinnamamide	C_9H_9NO	621-79-4	147.07	-0.00013
369	3-Methyl-1,2,3,4-tetrahydroisoquinoline	$C_{10}H_{13}N$	29726-60-1	147.10	-0.00021
370	(2E)-3-(3-Hydroxyphenyl)acrylaldehyde	$C_9H_8O_2$	26231-89-0	148.05	-0.00019

NO	Name	Formula	CAS	Molecular Weight (Da)	Annot.Delta Mass (Da)
371	1,3-Bis(2-Hydroxyethyl)urea	$C_5H_{12}N_2O_3$	15438-70-7	148.08	-0.00018
372	2-Ethyl-2-nitro-1,3-propanediol	$C_5H_{11}NO_4$	597-09-1	149.07	-0.00005
373	5-Formylsalicylaldehyde	$C_8H_6O_3$	3328-70-9	150.03	-0.00027
374	6-Hydroxy-3-coumaranone	$C_8H_6O_3$	6272-26-0	150.03	-0.00016
375	Vanillin	$C_8H_8O_3$	121-33-5	152.05	-0.00028
376	Methylparaben	$C_8H_8O_3$	99-76-3	152.05	-0.00024
377	Methyl salicylate	$C_8H_8O_3$	119-36-8	152.05	-0.00025
378	Resacetophenone	$C_8H_8O_3$	89-84-9	152.05	-0.00019
379	1-Adamantanol	$C_{10}H_{16}O$	768-95-6	152.12	-0.00025
380	N,N-Dimethyl-2-(2-methyl-1H-imi dazol-5-yl)ethanamine	$C_8H_{15}N_3$	ChemSpider ID 476026	153.13	-0.00036
381	Triacetonamine	$C_9H_{17}NO$	826-36-8	155.13	-0.00027
382	1-Benzylimidazole	$C_{10}H_{10}N_2$	4238-71-5	158.08	-0.00014
383	Methyl 2-morpholinoacetate	$C_7H_{13}NO_3$	35855-10-8	159.09	-0.00022
384	2-Morpholin-4-ylacetohydrazide	$C_6H_{13}N_3O_2$	770-17-2	159.10	-0.00013
385	4-Methoxycinnamaldehyde	$C_{10}H_{10}O_2$	1963-36-6	162.07	-0.00037
386	Methyl cinnamate	$C_{10}H_{10}O_2$	103-26-4	162.07	-0.00028
387	3’-Methoxypropiophenone	$C_{10}H_{12}O_2$	37951-49-8	164.08	-0.00034
388	Eugenol	$C_{10}H_{12}O_2$	97-53-0	164.08	-0.00027
389	Phloroacetophenone	$C_8H_8O_4$	480-66-0	168.04	-0.00025
390	D-3,4-Dihydroxyphenylglycolaldeh yde	$C_8H_8O_4$	52819-70-2	168.04	-0.00024
391	5-Formylfurfuryl acetate	$C_8H_8O_4$	10551-58-3	168.04	-0.00015
392	N-Methylcyclodecanamine	$C_{11}H_{23}N$	80789-66-8	169.18	-0.00023
393	Ethyl 4-amino-1-piperidinecarboxylate	$C_8H_{16}N_2O_2$	58859-46-4	172.12	-0.00017
394	tert-Butyl 2-hydroxyethylmethylcarbamate	$C_8H_{17}NO_3$	57561-39-4	175.12	-0.00016
395	5-Benzyldihydro-2(3H)-furanone	$C_{11}H_{12}O_2$	415902-37-3	176.08	-0.00036
396	Heptylbenzene	$C_{13}H_{20}$	1078-71-3	176.16	-0.00033
397	3-(1-Methylpyrrolidin-2-yl)pyridin-2-amine	$C_{10}H_{15}N_3$	32726-84-4	177.13	-0.00028
398	Allyl salicylate	$C_{10}H_{10}O_3$	10484-09-0	178.06	-0.00018
399	(E,E,E)-2,4,6-Dodecatrienal	$C_{12}H_{18}O$	147217-66-1	178.14	-0.00035
400	8-Hydroxy-6,7-dihydroisoquinoline -1,3(2H,5H)-dione	$C_9H_9NO_3$	37704-54-4	179.06	-0.00073
401	2-(6-Amino-1H-purin-1-yl)ethanol	$C_7H_9N_5O$	ChemSpider ID 58838095	179.08	-0.00029
402	4-Isopropyl-7-methyl-1H-azepine-2 ,5-dione	$C_{10}H_{13}NO_2$	10315-42-1	179.09	-0.00045
403	trans-Stilbene	$C_{14}H_{12}$	103-30-0	180.09	-0.00033
404	Methyl (E,E,Z)-2,4,6-Decatrienoate	$C_{11}H_{16}O_2$	51544-64-0	180.11	-0.00038

NO	Name	Formula	CAS	Molecular Weight (Da)	Annot.Delta Mass (Da)
405	2-tert-Butyl-4-methoxyphenol	$C_{11}H_{16}O_2$	121-00-6	180.11	-0.00038
406	2-(2-Methylenecyclopropyl)-3-oxosuccinate	$C_8H_6O_5$	ChemSpider ID 24785550	182.02	-0.00024
407	5-(2-Methylpropylidene)-2,4,6(1H,3H,5H)-pyrimidinetrione	$C_8H_{10}N_2O_3$	27406-43-5	182.07	-0.00051
408	Ethyl 4-hydroxy-2-methylpyrimidine-5-carboxylate	$C_8H_{10}N_2O_3$	53135-24-3	182.07	-0.00047
409	Mephenesin	$C_{10}H_{14}O_3$	59-47-2	182.09	-0.00026
410	10-Undecynoic acid	$C_{11}H_{18}O_2$	2777-65-3	182.13	-0.00029
411	(2E)-2-Tridecene-4,6,8-triyn-1-ol	$C_{13}H_{14}O$	6071-47-2	186.10	-0.00032
412	Methyl 1-acetyl-4-hydroxyprolinate	$C_8H_{13}NO_4$	ChemSpider ID 467588	187.08	-0.00014
413	Octahydro-1,6,7,8-indolizinetetrol	$C_8H_{15}NO_4$	142393-60-0	189.10	-0.00032
414	tert-Butyl (2-amino-2-thioxoethyl)carbamate	$C_7H_{14}N_2O_2S$	89226-13-1	190.08	-0.00033
415	2,6-Dimethoxy-4-allylphenol	$C_{11}H_{14}O_3$	6627-88-9	194.09	-0.00069
416	2-Octanoylfuran	$C_{12}H_{18}O_2$	5456-77-9	194.13	-0.00044
417	Sedanolide	$C_{12}H_{18}O_2$	6415-59-4	194.13	-0.0004
418	2-(Ethylamino)-4,5-dihydroxybenzamide	$C_9H_{12}N_2O_3$	127793-87-7	196.08	-0.00064
419	2-[2-(Benzyloxy)ethoxy]ethanol	$C_{11}H_{16}O_3$	2050-25-1	196.11	-0.00039
420	Dibenzylamine	$C_{14}H_{15}N$	103-49-1	197.12	-0.00038
421	2-Amino-2-[4-amino-6-(dimethylamino)-1,3,5-triazin-2-yl]ethanol	$C_7H_{14}N_6O$	ChemSpider ID 519465	198.12	0.00038
422	3,3-Dimethyl-1,5-dioxaspiro(5.5)undecan-9-one	$C_{11}H_{18}O_3$	69225-59-8	198.13	-0.00038
423	Ethyl 5-ethoxy-4-methyl-1,3-oxazole-2-carboxylate	$C_9H_{13}NO_4$	23429-04-1	199.08	-0.00029
424	(2Z)-2-Benzylidene-6-heptenal	$C_{14}H_{16}O$	ChemSpider ID 74853191	200.12	-0.00024
425	Diisopropyl diazene-1,2-dicarboxylate	$C_8H_{14}N_2O_4$	2446-83-5	202.10	-0.00032
426	Xanthurenic acid	$C_{10}H_7NO_4$	59-00-7	205.04	-0.00031
427	Ethyl methylphenylglycidate	$C_{12}H_{14}O_3$	77-83-8	206.09	-0.00037
428	Asarone	$C_{12}H_{16}O_3$	2883-98-9	208.11	-0.00024
429	2-Methylene-5,10-undecadienoic acid methyl ester	$C_{13}H_{20}O_2$	51788-60-4	208.15	-0.00042
430	4-Heptyloxyphenol	$C_{13}H_{20}O_2$	13037-86-0	208.15	-0.00033
431	5-(4-Methoxyphenyl)-1,3-cyclohexanedione	$C_{13}H_{14}O_3$	1774-12-5	218.09	-0.00042

NO	Name	Formula	CAS	Molecular Weight (Da)	Annot.Delta Mass (Da)
432	(4E)-6-(1,3-Benzodioxol-5-yl)-4-hexen-3-one	$C_{13}H_{14}O_3$	ChemSpider ID 4509686	218.09	-0.00039
433	D-Pantothenic acid	$C_9H_{17}NO_5$	79-83-4	219.11	-0.00033
434	1-(5-Acetyl-2-hydroxyphenyl)-3-methyl-1-butanone	$C_{13}H_{16}O_3$	62458-64-4	220.11	-0.00045
435	Hexamethylcyclotrisiloxane	$C_6H_{18}O_3Si_3$	541-05-9	222.06	-0.00044
436	Methyl 3-(5-oxo-4,5,6,7-tetrahydro-1H-pyrrolo[2,3-c]pyridin-3-yl)propanoate	$C_{11}H_{14}N_2O_3$	ChemSpider ID 533528	222.10	-0.00037
437	N-[2-(4-Morpholinyl)ethyl]-1H-1,2,4-triazole-5-carboxamide	$C_9H_{15}N_5O_2$	ChemSpider ID 493565	225.12	-0.0005
438	1-[(2,2-Dimethyl-1,3-dioxolan-4-yl)methyl]-2,4(1H,3H)-pyrimidinedione	$C_{10}H_{14}N_2O_4$	ChemSpider ID 3233081	226.09	-0.00045
439	1,6-Hexanediol diacrylate	$C_{12}H_{18}O_4$	13048-33-4	226.12	-0.00052
440	1,8-Diazacyclotetradecan-2,9-dion	$C_{12}H_{22}N_2O_2$	56403-09-9	226.17	-0.00034
441	Dimethyl 1,4-diazabicyclo[2.2.2]octane-2,3-dicarboxylate	$C_{10}H_{16}N_2O_4$	ChemSpider ID 519583	228.11	-0.00019
442	5-Hydroxy-6-methoxy-7H-furo[3,2-g]chromen-7-one	$C_{12}H_8O_5$	35779-46-5	232.04	-0.00036
443	4-(4-Hydroxy-3-methoxyphenyl)-3,6-dihydro-1(2H)-pyridinecarbaldehyde	$C_{13}H_{15}NO_3$	ChemSpider ID 535604	233.10	-0.00064
444	3-Hydroxy-4,5,6-trimethoxytetrahydro-2H-pyran-2-carboxamide	$C_9H_{17}NO_6$	ChemSpider ID 482310	235.11	-0.00048
445	4-Hydroxy-4-(5-methoxy-2-nitrophenyl)-2-butanone	$C_{11}H_{13}NO_5$	681446-84-4	239.08	-0.00052
446	2,5-Anhydro-1-deoxy-1-(2-pyridinylamino)hexitol	$C_{11}H_{16}N_2O_4$	ChemSpider ID 497379	240.11	-0.00036
447	4,5-Epoxymorphinan	$C_{16}H_{19}NO$	ChemSpider ID 536950	241.15	-0.00066
448	Methyl 2-[(cyclohexylcarbonyl)amino]-3-methylbutanoate	$C_{13}H_{23}NO_3$	ChemSpider ID 479972	241.17	-0.0006
449	1-(2,4-Dideoxyhexopyranosyl)-2,4(1H,3H)-pyrimidinedione	$C_{10}H_{14}N_2O_5$	ChemSpider ID 506602	242.09	-0.00043
450	Dimethyl N-isobutyrylglutamate	$C_{11}H_{19}NO_5$	ChemSpider ID 469496	245.13	-0.00042
451	2,3’,4,6-Tetrahydroxybenzophenone	$C_{13}H_{10}O_5$	26271-33-0	246.05	-0.00047
452	2-(12-Tridecyn-1-yl)furan	$C_{17}H_{26}O$	24708-33-6	246.20	-0.00046

NO	Name	Formula	CAS	Molecular Weight (Da)	Annot.Delta Mass (Da)
453	Podocarpan-12-ol	$C_{17}H_{30}O$	ChemSpider ID 503994	250.23	-0.00072
454	6-Hydroxy-4,4,7-trimethyl-5-nitro-2-chromanone	$C_{12}H_{13}NO_5$	ChemSpider ID 538525	251.08	-0.00066
455	Mono(3-carboxypropyl) phthalate	$C_{12}H_{12}O_6$	66851-46-5	252.06	-0.00052
456	(2Z)-3-(6,7-Dimethoxy-1,3-benzodioxol-5-yl)-2-hydroxyacrylaldehyde	$C_{12}H_{12}O_6$	ChemSpider ID 74852295	252.06	-0.00035
457	Pirimicarb-desmethyl-formamido	$C_{11}H_{16}N_4O_3$	27218-04-8	252.12	-0.00069
458	2-[4-(3-Hydroxypropyl)-2-methoxy phenoxy]-1,3-propanediol	$C_{13}H_{20}O_5$	71046-09-8	256.13	-0.0007
459	Octadecapenten-3-one	$C_{18}H_{26}O$	ChemSpider ID 10476703	258.20	-0.00063
460	Propyl N-(methoxycarbonyl)-N-methylglycyl-N-methylglycinate	$C_{11}H_{20}N_2O_5$	ChemSpider ID 4927395	260.14	-0.00046
461	Maclurin	$C_{13}H_{10}O_6$	519-34-6	262.05	-0.00052
462	Methohexital	$C_{14}H_{18}N_2O_3$	151-83-7	262.13	-0.00058
463	1-(2-Methoxy-5-nitrobenzyl)-4-methylpiperazine	$C_{13}H_{19}N_3O_3$	ChemSpider ID 471676	265.14	-0.0004
464	Ethyl (2Z)-[(1,3-benzodioxol-5-ylmethyl) imino](hydroxyamino)acetate	$C_{12}H_{14}N_2O_5$	ChemSpider ID 4911614	266.09	-0.00048
465	(2E)-N-(4-Amino-2,3-dihydroxybutyl)-3-(4-hydroxyphenyl)acrylamide	$C_{13}H_{18}N_2O_4$	ChemSpider ID 59650572	266.13	-0.00056
466	Methyl 2-hydroxy-6-methyl-3-nitro-4-(2-oxopropyl)benzoate	$C_{12}H_{13}NO_6$	89586-50-5	267.07	-0.00046
467	Rhamnopterin	$C_{10}H_{13}N_5O_4$	13392-24-0	267.10	-0.00057
468	1-Decanoyl-2,6-dimethylpiperidine	$C_{17}H_{33}NO$	4629-19-0	267.26	-0.00075
469	Thiodiacetophenone	$C_{16}H_{14}O_2S$	2461-80-5	270.07	-0.00075
470	(3E)-3-[(Methoxyacetyl)hydrazono]-N-(tetrahydro-2-furanylmethyl)butanamide	$C_{12}H_{21}N_3O_4$	ChemSpider ID 7871039	271.15	-0.00085
471	Di-2-pentanyl malate	$C_{14}H_{26}O_5$	ChemSpider ID 468884	274.18	0.00053
472	4,5,6-Trimethoxy-7H-furo[3,2-g]chromen-7-one	$C_{14}H_{12}O_6$	18646-71-4	276.06	-0.00078
473	p-Coumaroylagmatine	$C_{14}H_{20}N_4O_2$	7295-86-5	276.16	-0.00059
474	6-Shogaol	$C_{17}H_{24}O_3$	555-66-8	276.17	-0.00061
475	Phenyl laurate	$C_{18}H_{28}O_2$	4228-00-6	276.21	-0.00081
476	[3-Acetyloxy-2-(acetyloxymethyl)-2-nitropropyl] acetate	$C_{10}H_{15}NO_8$	7344-23-2	277.08	-0.00091

NO	Name	Formula	CAS	Molecular Weight (Da)	Annot.Delta Mass (Da)
477	6,8-Dihydroxy-3-[(2S)-2-hydroxy-4-oxopentyl]-1H-isochromen-1-one	$C_{14}H_{14}O_6$	ChemSpider ID 31143389	278.08	-0.00062
478	4-(3-Butoxy-4-methoxybenzyl)-2-imidazolidinone	$C_{15}H_{22}N_2O_3$	29925-17-5	278.16	-0.00068
479	Heptyl (4-nitrophenyl)carbamate; Heptyl 4-nitrophenylcarbamate	$C_{14}H_{20}N_2O_4$	92374-99-7	280.14	-0.0006
480	(2,2-Dimethyl-1,3-dioxolan-4-yl)methyl 4-nitrobenzoate	$C_{13}H_{15}NO_6$	ChemSpider ID 470054	281.09	-0.00081
481	N,N'-(2,4,5-Trimethoxy-1,3-phenylene)diacetamide	$C_{13}H_{18}N_2O_5$	ChemSpider ID 524502	282.12	-0.00051
482	N-Acetoxy-N-[5-(2,5-dioxo-1-pyrrolidinyl)pentyl]acetamide	$C_{13}H_{20}N_2O_5$	ChemSpider ID 470427	284.14	-0.00045
483	(2Z)-4,6,7-Trihydroxy-2-(4-hydroxybenzylidene)-1-benzofuran-3(2H)-one	$C_{15}H_{10}O_6$	ChemSpider ID 24846195	286.05	-0.00072
484	2,5-Dioxo-1-pyrrolidinyl N-{[(2-methyl-2-propanyl)oxy]carbonyl}alaninate	$C_{12}H_{18}N_2O_6$	3392-05-0	286.12	-0.00079
485	Diethyl [amino(2-isobutyrylhydrazino)methylene]malonate	$C_{12}H_{21}N_3O_5$	ChemSpider ID 1651360	287.15	-0.00072
486	2-[(4-Amino-7H-pyrrolo[2,3-d]pyrimidin-7-yl)methoxy]ethyl dihydrogen phosphate	$C_9H_{13}N_4O_5P$	86626-01-9	288.06	0.00047
487	1,5-Anhydro-1-(2,4,6-trihydroxyphenyl)hexitol	$C_{12}H_{16}O_8$	ChemSpider ID 74886754	288.08	-0.0007
488	Pentyl N-(methoxycarbonyl)-N-methylglycyl-N-methylglycinate	$C_{13}H_{24}N_2O_5$	ChemSpider ID 4927397	288.17	-0.0009
489	6-Amino-1-[2-(3,4-dimethoxyphenyl)ethyl]pyrimidine-2,4(1H,3H)-dione	$C_{14}H_{17}N_3O_4$	446266-76-8	291.12	-0.00061
490	2-Acetyl-4,7,8-trihydroxy-6-methoxy-3-methyl-1,5-naphthalenedione	$C_{14}H_{12}O_7$	1228-77-9	292.06	-0.00039
491	Methyl alpha-aspartylphenylalaninate	$C_{14}H_{18}N_2O_5$	22839-65-2	294.12	-0.00065
492	Troxipide	$C_{15}H_{22}N_2O_4$	30751-05-4	294.16	-0.00061
493	Disulfiram	$C_{10}H_{20}N_2S_4$	97-77-8	296.05	-0.00066
494	Octamethylcyclotetrasiloxane	$C_8H_{24}O_4Si_4$	556-67-2	296.07	-0.00051
495	2,3,5,6-Tetrahydroxy-4-methoxy-N'-phenylhexanehydrazide	$C_{13}H_{20}N_2O_6$	7404-33-3	300.13	-0.00041
496	3-Hydroxy-cis-5-octenoylcarnitine	$C_{15}H_{27}NO_5$	ChemSpider ID	301.19	-0.00082

NO	Name	Formula	CAS	Molecular Weight (Da)	Annot.Delta Mass (Da)
			28639168		
497	1,5-Dicyclopentyl-3-(2-cyclopentyl -ethyl)-2-pentene	$C_{22}H_{38}$	54934-71-3	302.30	-0.00076
498	3-(2,4,5-Trihydroxyphenyl)-4,6,7-c hromanetriol	$C_{15}H_{14}O_7$	ChemSpider ID 74851598	306.07	-0.00076
499	Myristic acid diethanolamide	$C_{18}H_{37}NO_3$	7545-23-5	315.28	-0.00063
500	1-(4-Ethoxyphenyl)-3-(4-methyl-1-piperazinyl)-2,5-pyrrolidinedione	$C_{17}H_{23}N_3O_3$	21621-46-5	317.17	-0.00071
501	2-Aminooctadecane-1,3,4-triol	$C_{18}H_{39}NO_3$	13552-11-9	317.29	-0.00073
502	Methyl N-[1-(4-ethoxyphenyl)-2,5-dioxo-3-pyrrolidinyl]alaninate	$C_{16}H_{20}N_2O_5$	1008942-96-8	320.14	-0.00076
503	2-sec-Butyl-4,6-dinitrophenyl isopropyl carbonate	$C_{14}H_{18}N_2O_7$	973-21-7	326.11	-0.00071
504	Isopropyl 10-hydroxy-11-(4-morpholinyl)und ecanoate	$C_{18}H_{35}NO_4$	ChemSpider ID 3235787	329.26	-0.00098
505	(2S,3S)-2-(Dimethylamino)-1,3-oct adecanediol	$C_{20}H_{43}NO_2$	ChemSpider ID 24823237	329.33	-0.00084
506	2-[4-(Benzyloxy)-3-ethoxyphenyl]-2-hydroxyethyl acetate	$C_{19}H_{22}O_5$	ChemSpider ID 490054	330.15	-0.00091
507	Dimethyl 4-(2,5-dimethoxyphenyl)-1,4-dihyd ro-3,5-pyridinedicarboxylate	$C_{17}H_{19}NO_6$	ChemSpider ID 527848	333.12	-0.00084
508	N-Benzyl-N-isopropyl-2-naphthale nesulfonamide	$C_{20}H_{21}NO_2S$	ChemSpider ID 643051	339.13	0.00158
509	Methyl 6-(dimethylamino)octadecanoate	$C_{21}H_{43}NO_2$	56817-90-4	341.33	-0.00102
510	2,2’-(3,6-Dioxo-2,5-piperazinediyl) bis(N-hydroxy-N-propylacetamide)	$C_{14}H_{24}N_4O_6$	ChemSpider ID 477146	344.17	-0.0009
511	3-[(3-Hydroxyundecanoyl)oxy]-4-(t rimethylammonio)butanoate	$C_{18}H_{35}NO_5$	ChemSpider ID 59664326	345.25	-0.00111
512	3-Methyl-5-(5,5,8a-trimethyl-2-met hylene-7-oxodecahydro-1-naphthale nyl)pentyl acetate	$C_{22}H_{36}O_3$	ChemSpider ID 29814842	348.27	-0.00062
513	10-Undecenyl-1-N-(cyclohexylcarb onyl)alaninate	$C_{21}H_{37}NO_3$	ChemSpider ID 4926097	351.28	-0.00081
514	Tridecyl N-[(allyloxy)carbonyl]alaninate	$C_{20}H_{37}NO_4$	ChemSpider ID 4928205	355.27	-0.00103
515	Tetradecyl N-(ethoxycarbonyl)alaninate	$C_{20}H_{39}NO_4$	ChemSpider ID 4927832	357.29	-0.00107
516	3-(7-Hydroxy-4b-methyl-2-methyle	$C_{25}H_{34}O_2$	ChemSpider ID	366.26	-0.00079

NO	Name	Formula	CAS	Molecular Weight (Da)	Annot.Delta Mass (Da)
	netetradecahydro-1-phenanthrenyl)-1-phenyl-1-propanone		537775		
517	Decamethylcyclopentasiloxane	$C_{10}H_{30}O_5Si_5$	541-02-6	370.09	-0.00091
518	Riboflavin	$C_{17}H_{20}N_4O_6$	83-88-5	376.14	-0.00113
519	1,6-Dimethyl 2-O-(3,4,5-trihydroxybenzoyl)hexarate	$C_{15}H_{18}O_{12}$	ChemSpider ID 35014325	390.08	-0.00065
520	Tetramethyl 1-tert-butyl-1,6-dihydro-6-methoxy-2,3,4,5-pyridinetetracarboxylate	$C_{18}H_{25}NO_9$	ChemSpider ID 538592	399.15	-0.00092
521	3-Methoxy-1,2-didehydrocrinan-6,11-diyl diacetate	$C_{21}H_{23}NO_7$	ChemSpider ID 470632	401.15	-0.00109
522	Octyl propyl undecyl phosphate	$C_{22}H_{47}O_4P$	ChemSpider ID 4927331	406.32	0.00127
523	7-Dehydrocholesterol acetate	$C_{29}H_{46}O_2$	1059-86-5	426.35	-0.00099
524	Cholesta-7,14-dien-7-yl acetate	$C_{29}H_{46}O_2$	ChemSpider ID 549949	426.35	-0.00071
525	(3beta,5beta,6beta)-5,6-Epoxyergost-24(28)-ene-3,19-diol	C28H46O3	ChemSpider ID 10283126	430.34	-0.00101
526	Trimazosin	$C_{20}H_{29}N_5O_6$	35795-16-5	435.21	-0.00202
527	Oleanolic aldehyde	$C_{30}H_{48}O_2$	17020-22-3	440.36	-0.0011
528	7-Hydroxycholest-5-en-3-yl benzoate	$C_{34}H_{50}O_3$	40824-59-7	506.37	-0.00112
529	Pheophorbide A	$C_{35}H_{36}N_4O_5$	15664-29-6	592.27	-0.00176

Note: Annot. Delta Mass represents the difference between the actual measured value of molecular weight and the theoretical value.

Table S2. List of 349 non-volatile metabolites in the processing of Rizhao green tea (ESI−, C18 column).

NO	Name	Formula	CAS	Molecular Weight (Da)	Annot.Delta Mass (Da)
1	N-Carbobenzoxy-DL-serine	$C_{11}H_{13}NO_5$	2768-56-1	239.08	0.00003
2	Val-Val	$C_{10}H_{20}N_2O_3$	3918-94-3	216.15	0.00036
3	N-Acetylvaline	$C_7H_{13}NO_3$	3067-19-4	159.09	0.00004
4	N-Acetyl-DL-tryptophan	$C_{13}H_{14}N_2O_3$	87-32-1	246.10	-0.00048
5	N-Acetyl-DL-phenylalanine	$C_{11}H_{13}NO_3$	2901-75-9	207.09	-0.00024
6	N-Acetyl-L-leucine	$C_8H_{15}NO_3$	1188-21-2	173.11	-0.00002
7	N6-((Benzyloxy)carbonyl)lysine	$C_{14}H_{20}N_2O_4$	32302-83-3	280.14	0.00053
8	N2-(tert-Butoxycarbonyl)glutamin	$C_{10}H_{18}N_2O_5$	85535-45-1	246.12	-0.00017
9	Boc-4-Nitro-L-phenylalanine	$C_{14}H_{18}N_2O_6$	33305-77-0	310.12	-0.00009
10	DL-Alanyl-DL-leucine	$C_9H_{18}N_2O_3$	1638-60-4	202.13	-0.00001
11	alpha-Methyl-L-tyrosine	$C_{10}H_{13}NO_3$	672-87-7	195.09	-0.00018
12	L-Theanine	$C_7H_{14}N_2O_3$	3081-61-6	174.10	-0.00045
13	L-gamma-Glutamyl-L-leucine	$C_{11}H_{20}N_2O_5$	2566-39-4	260.14	0.00008
14	Leu-Val	$C_{11}H_{22}N_2O_3$	35436-83-0	230.16	-0.00016
15	Leu-pro	$C_{11}H_{20}N_2O_3$	6403-35-6	228.15	0.00033
16	Leu-Leu	$C_{12}H_{24}N_2O_3$	3303-31-9	244.18	0.00009
17	Leu-Gly-Gly	$C_{10}H_{19}N_3O_4$	4337-37-5	245.14	0.00014
18	Hexahydrohippuric acid	$C_9H_{15}NO_3$	32377-88-1	185.10	-0.00049
19	Gly-Phe	$C_{11}H_{14}N_2O_3$	721-66-4	222.10	0.00031
20	Gly-Leu	$C_8H_{16}N_2O_3$	869-19-2	188.12	0.00033
21	DL-Tryptophan	$C_{11}H_{12}N_2O_2$	54-12-6	204.09	-0.00040
22	DL-Phenylalanine	$C_9H_{11}NO_2$	150-30-1	165.08	-0.00008
23	Dihydroxyphenylalanine	$C_9H_{11}NO_4$	63-84-3	197.07	-0.00016
24	Boc-Glycine	$C_7H_{13}NO_4$	4530-20-5	175.08	0.00016
25	Ala-Tyr	$C_{12}H_{16}N_2O_4$	3061-88-9	252.11	0.00018
26	5-Hydroxy-DL-tryptophan	$C_{11}H_{12}N_2O_3$	56-69-9	220.08	-0.00004
27	2-Morpholinoacetic acid	$C_6H_{11}NO_3$	3235-69-6	145.07	-0.00011
28	1-(tert-Butoxycarbonyl)proline	$C_{10}H_{17}NO_4$	59433-50-0	215.12	0.00012
29	1-(Ethoxycarbonyl)proline	$C_8H_{13}NO_4$	5700-74-3	187.08	0.00007
30	Methyl gallate	$C_8H_8O_5$	99-24-1	184.04	-0.00058
31	Ellagic acid	$C_{14}H_6O_8$	476-66-4	302.01	-0.00063
32	Procyanidin B2	$C_{30}H_{26}O_{12}$	29106-49-8	578.14	-0.00124
33	Gallic acid	$C_7H_6O_5$	149-91-7	170.02	-0.00041
34	Digallic acid	$C_{14}H_{10}O_9$	536-08-3	322.03	-0.00046
35	Epigallocatechin 3-O-(3-O-methyl)gallate	$C_{23}H_{20}O_{11}$	83104-87-4	472.10	-0.00089
36	(-)-Epigallocatechin 3'-O-glucuronide	$C_{21}H_{22}O_{13}$	569670-41-3	482.11	-0.00003
37	(+)-Gallocatechin	$C_{15}H_{14}O_7$	970-73-0	306.07	-0.00074
38	(-)-Epigallocatechin gallate	$C_{22}H_{18}O_{11}$	989-51-5	458.08	-0.00118

NO	Name	Formula	CAS	Molecular Weight (Da)	Annot.Delta Mass (Da)
39	(-)-Epiafzelechin 3-O-gallate	$C_{22}H_{18}O_9$	108907-43-3	426.09	-0.00052
40	Epicatechin 3-O-(3-O-methylgallate)	$C_{23}H_{20}O_{10}$	83104-86-3	456.11	-0.00029
41	(-)-Epicatechin gallate	$C_{22}H_{18}O_{10}$	1257-08-5	442.09	-0.00131
42	Epitheaflagallin 3-O-gallate	$C_{27}H_{20}O_{13}$	102067-92-5	552.09	-0.00008
43	Methylgallic acid-O-sulphate	$C_8H_8O_8S$	ChemSpider ID 30778507	263.99	0.00020
44	5-Sulfosalicylic acid	$C_7H_6O_6S$	97-05-2	217.99	0.00005
45	Sinapinic acid	$C_{11}H_{12}O_5$	530-59-6	224.07	-0.00033
46	Salicylic acid	$C_7H_6O_3$	69-72-7	138.03	-0.00067
47	Protocatechuic acid	$C_7H_6O_4$	99-50-3	154.03	-0.00054
48	Coniferyl ferulate	$C_{20}H_{20}O_6$	63644-62-2	356.13	0.00005
49	Chlorogenic acid	$C_{16}H_{18}O_9$	327-97-9	354.09	-0.00043
50	Caftaric acid	$C_{13}H_{12}O_9$	67879-58-7	312.05	0.00030
51	3,4-Dihydroxyphenylpyruvic acid	$C_9H_8O_5$	4228-66-4	196.04	-0.00011
52	3,4-Dihydroxyphenylacetic acid	$C_8H_8O_4$	102-32-9	168.04	-0.00013
53	Theaflavic acid	$C_{21}H_{16}O_{10}$	30407-93-3	428.07	-0.00066
54	2-Protocatechuoyl phloroglucinolcarboxylate	$C_{14}H_{10}O_8$	30048-34-1	306.04	-0.00021
55	2-Caffeoylisocitric acid	$C_{15}H_{14}O_{10}$	ChemSpider ID 4444181	354.06	-0.00030
56	(+)-Chebulic acid	$C_{14}H_{12}O_{11}$	ChemSpider ID 35014171	356.04	-0.00029
57	4-Hydroxycinnamic acid	$C_9H_8O_3$	501-98-4	164.05	-0.00029
58	Xanthosine	$C_{10}H_{12}N_4O_6$	146-80-5	284.08	-0.00001
59	Thymidine	$C_{10}H_{14}N_2O_5$	50-89-5	242.09	-0.00031
60	Succinyladenosine	$C_{14}H_{17}N_5O_8$	4542-23-8	383.11	0.00035
61	N-Acetylcytidine	$C_{11}H_{15}N_3O_6$	3768-18-1	285.10	0.00030
62	1-Methylxanthine	$C_6H_6N_4O_2$	6136-37-4	166.05	-0.00018
63	Guanine	$C_5H_5N_5O$	73-40-5	151.05	-0.00022
64	Deoxyuridine	$C_9H_{12}N_2O_5$	951-78-0	228.07	0.00024
65	Deoxyinosine	$C_{10}H_{12}N_4O_4$	890-38-0	252.09	-0.00003
66	Cyclic di-AMP	$C_{20}H_{24}N_{10}O_{12}P_2$	54447-84-6	658.10	-0.00014
67	c-GMP-AMP	$C_{20}H_{24}N_{10}O_{13}P_2$	849214-04-6	674.10	-0.00007
68	Arabinosylhypoxanthine	$C_{10}H_{12}N_4O_5$	7013-16-3	268.08	-0.00001
69	Adenine	$C_5H_5N_5$	73-24-5	135.05	-0.00005
70	8-Hydroxy-2'-deoxyguanosine	$C_{10}H_{13}N_5O_5$	88847-89-6	283.09	-0.00041
71	5-Methyluridine	$C_{10}H_{14}N_2O_6$	1463-10-1	258.09	0.00000
72	Theogallin	$C_{14}H_{16}O_{10}$	17365-11-6	344.07	-0.00125
73	Theasinensin A	$C_{44}H_{34}O_{22}$	89064-31-3	914.15	-0.00056

NO	Name	Formula	CAS	Molecular Weight (Da)	Annot.Delta Mass (Da)
74	Taxifolin	$C_{15}H_{12}O_7$	480-18-2	304.06	-0.00020
75	Swertianolin	$C_{20}H_{20}O_{11}$	23445-00-3	436.10	-0.00017
76	Scutellarin	$C_{21}H_{18}O_{12}$	27740-01-8	462.08	0.00002
77	Apigenin-7-O-rhamnoglucoside	$C_{27}H_{30}O_{14}$	17306-46-6	578.16	-0.00118
78	Quercitrin	$C_{21}H_{20}O_{11}$	522-12-3	448.10	-0.00100
79	Naringenin-7-O-glucoside	$C_{21}H_{22}O_{10}$	529-55-5	434.12	-0.00049
80	Plantagoside	$C_{21}H_{22}O_{12}$	78708-33-5	466.11	-0.00065
81	Kaempferol	$C_{15}H_{10}O_6$	520-18-3	286.05	-0.00023
82	3',4',5,7-Tetrahydroxyflavanone	$C_{15}H_{12}O_6$	4049-38-1	288.06	0.00019
83	Cianidanol	$C_{15}H_{14}O_6$	154-23-4	290.08	-0.00078
84	5,7,3',4'-Tetrahydroxy-3,6,8-trimeth oxyflavone	$C_{18}H_{16}O_9$	61451-85-2	376.08	-0.00057
85	3,4,5,7,3’,4’,5’-Heptahydroxyflavan	$C_{15}H_{14}O_8$	491-52-1	322.07	-0.00084
86	4’,5,7-Trihydroxyflavanone	$C_{15}H_{12}O_5$	67604-48-2	272.07	-0.00039
87	Phloretin	$C_{15}H_{14}O_5$	60-82-2	274.08	-0.00009
88	6-Methoxyquercetin	$C_{16}H_{12}O_8$	519-96-0	332.05	-0.00014
89	Kaempferol-3-O-rutinoside	$C_{27}H_{30}O_{15}$	17650-84-9	594.16	-0.00128
90	Miquelianin	$C_{21}H_{18}O_{13}$	22688-79-5	478.07	-0.00079
91	Kaempferol 3-arabinofuranoside	$C_{20}H_{18}O_{10}$	5041-67-8	418.09	-0.00063
92	Isoquercetin	$C_{21}H_{20}O_{12}$	482-35-9	464.09	-0.00078
93	Salvigenin	$C_{18}H_{16}O_6$	19103-54-9	328.09	-0.00038
94	Gossypin	$C_{21}H_{20}O_{13}$	652-78-8	480.09	-0.00086
95	Eucalyptin	$C_{19}H_{18}O_5$	3122-88-1	326.12	-0.00018
96	Dihydromyricetin	$C_{15}H_{12}O_8$	27200-12-0	320.05	-0.00025
97	Baicalin	$C_{21}H_{18}O_{11}$	21967-41-9	446.08	-0.00037
98	Quercetin 3-O-alpha-L-arabinopyranoside	$C_{20}H_{18}O_{11}$	572-30-5	434.08	-0.00033
99	Taxifolin 3-O-rhamnoside	$C_{21}H_{22}O_{11}$	29838-67-3	450.12	-0.00053
100	5,7-Dihydroxy-3’,4’,5’-trimethoxyf lavanone	$C_{18}H_{18}O_7$	62252-10-2	346.11	0.00011
101	Scoparone	$C_{11}H_{10}O_4$	120-08-1	206.06	-0.00033
102	4-Methylumbelliferyl-beta-D-glucu ronide	$C_{16}H_{16}O_9$	6160-80-1	352.08	0.00029
103	4-Methylumbelliferyl-beta-D-gluco pyranoside	$C_{16}H_{18}O_8$	18997-57-4	338.10	-0.00089
104	7-Ethoxy-4-methylcoumarin	$C_{12}H_{12}O_3$	87-05-8	204.08	-0.00007
105	4-Hydroxycoumarin	$C_9H_6O_3$	1076-38-6	162.03	-0.00029
106	Coumarin	$C_9H_6O_2$	91-64-5	146.04	0.00014
107	Aesculin	$C_{15}H_{16}O_9$	531-75-9	340.08	0.00002
108	7-Ethoxycoumarin	$C_{11}H_{10}O_3$	31005-02-4	190.06	-0.00011
109	4-Methyl-6,7-diacetoxycoumarin	$C_{14}H_{12}O_6$	55939-28-1	276.06	-0.00016
110	4,5,7-Trihydroxycoumarin	$C_9H_6O_5$	17575-26-7	194.02	-0.00027

NO	Name	Formula	CAS	Molecular Weight (Da)	Annot.Delta Mass (Da)
111	Vanillin	$C_8H_8O_3$	121-33-5	152.05	-0.00053
112	Trimethyl 4-hydroxy-5-methyl-1,2,3-benzenet ricarboxylate	$C_{13}H_{14}O_7$	ChemSpider ID 538404	282.07	-0.00084
113	Triethyl 1,3,5-benzenetricarboxylate	$C_{15}H_{18}O_6$	4105-92-4	294.11	0.00018
114	4-(3-Butoxy-4-methoxybenzyl)-2-i midazolidinone	$C_{15}H_{22}N_2O_3$	29925-17-5	278.16	0.00033
115	Pyrogallol	$C_6H_6O_3$	87-66-1	126.03	-0.00011
116	Oxydi-3,1,2-propanetriyl tetrabutanoate	$C_{22}H_{38}O_9$	ChemSpider ID 479813	446.25	-0.00022
117	N-(5-Phenyl-1H-1,2,4-triazol-3-yl)b enzamide	$C_{15}H_{12}N_4O$	ChemSpider ID 495787	264.10	-0.00121
118	Monobutyrin	$C_7H_{14}O_4$	557-25-5	162.09	0.00005
119	6-Methyl-5-hepten-2-one	$C_8H_{14}O$	110-93-0	126.10	0.00009
120	Lauric anhydride	$C_{24}H_{46}O_3$	645-66-9	382.35	0.00057
121	Methyl p-toluenesulfonate	$C_8H_{10}O_3S$	80-48-8	186.04	0.00026
122	Methyl alpha-aspartylphenylalaninate	$C_{14}H_{18}N_2O_5$	22839-65-2	294.12	0.00064
123	Methyl 3-hydroxydodecanoate	C13H26O3	72864-23-4	230.19	0.00011
124	Methyl 3,5-di-tert-butyl-4-hydroxybenzoate	$C_{16}H_{24}O_3$	2511-22-0	264.17	0.00011
125	Maclurin	$C_{13}H_{10}O_6$	519-34-6	262.05	0.00010
126	Indole	C_8H_7N	120-72-9	117.06	-0.00004
127	Hexyl fumarate	$C_{16}H_{28}O_4$	19139-31-2	284.20	0.00022
128	Heptanoic anhydride	$C_{14}H_{26}O_3$	626-27-7	242.19	0.00016
129	Guaiacol sulfate	$C_7H_8O_5S$	3233-59-8	204.01	0.00008
130	Guaiacol	$C_7H_8O_2$	544-31-0	124.05	0.00025
131	Diethylene glycol monolaurate	$C_{16}H_{32}O_4$	141-20-8	288.23	0.00008
132	2-Hydroxyquinoline	C_9H_7NO	59-31-4	145.05	0.00012
133	Ethyl p-hydroxybenzoate	$C_9H_{10}O_3$	120-47-8	166.06	0.00009
134	Ethylene dodecanedioate	$C_{14}H_{24}O_4$	54982-83-1	256.17	0.00013
135	Ethyl lactate	$C_5H_{10}O_3$	97-64-3	118.06	-0.00044
136	Ethyl cinnamate	$C_{11}H_{12}O_2$	103-36-6	176.08	0.00008
137	Ethyl 2-oxocyclohexanecarboxylate	$C_9H_{14}O_3$	1655-07-8	170.09	-0.00009
138	Ethyl 2-acetylheptanoate	$C_{11}H_{20}O_3$	24317-94-0	200.14	-0.00009
139	Epicatechin-3'-sulfate	$C_{15}H_{14}O_9S$	1038922-77-8	370.04	0.00006
140	6-Nitroveratraldehyde	$C_9H_9NO_5$	20357-25-9	211.05	0.00004
141	Di-tert-butyl malonate	$C_{11}H_{20}O_4$	541-16-2	216.14	0.00017
142	Diphenol glucuronide	$C_{12}H_{14}O_8$	ChemSpider ID 30778502	286.07	-0.00028
143	Dimethyl itaconate	$C_7H_{10}O_4$	617-52-7	158.06	-0.00014

NO	Name	Formula	CAS	Molecular Weight (Da)	Annot.Delta Mass (Da)
144	Dimethyl 2,3-bis(1-methoxyethoxy)succinate	$C_{12}H_{22}O_8$	ChemSpider ID 476915	294.13	0.00003
145	Dimethyl 1,4-cyclohexanedicarboxylate	$C_{10}H_{16}O_4$	94-60-0	200.10	-0.00011
146	Dimedone	$C_8H_{12}O_2$	126-81-8	140.08	0.00004
147	Diethyl tartrate	$C_8H_{14}O_6$	13811-71-7	206.08	-0.00013
148	Diethyl phosphate	$C_4H_{11}O_4P$	598-02-7	154.04	0.00013
149	Diethyl malonate	$C_7H_{12}O_4$	105-53-3	160.07	0.00011
150	Diethyl benzoylmalonate	$C_{14}H_{16}O_5$	1087-97-4	264.10	0.00003
151	Dibutyl malate	$C_{12}H_{22}O_5$	6280-99-5	246.15	-0.00015
152	Dibutyl itaconate	$C_{13}H_{22}O_4$	2155-60-4	242.15	-0.00008
153	Dibutyl Fumarate	$C_{12}H_{20}O_4$	105-75-9	228.14	-0.00007
154	Ethyl p-methoxycinnamate	$C_{12}H_{14}O_3$	24393-56-4	206.09	0.00017
155	Diallyl phthalate	$C_{14}H_{14}O_4$	131-17-9	246.09	-0.00053
156	Coumarone	C_8H_6O	271-89-6	118.04	-0.00017
157	Caproic anhydride	$C_{12}H_{22}O_3$	2051-49-2	214.16	-0.00010
158	Butylphthalide	$C_{12}H_{14}O_2$	6066-49-5	190.10	-0.00011
159	Butyl acrylate	$C_7H_{12}O_2$	141-32-2	128.08	0.00000
160	Bis(2-methoxyethyl) tartarate	$C_{10}H_{18}O_8$	ChemSpider ID 476785	266.10	-0.00015
161	Bis(2-butoxyethyl) adipate	$C_{18}H_{34}O_6$	141-18-4	346.24	-0.00035
162	Benzoquinone	$C_6H_4O_2$	106-51-4	108.02	0.00006
163	Phenol	C_6H_6O	108-95-2	94.04	0.00001
164	Bellidifolin	$C_{14}H_{10}O_6$	2798-25-6	274.05	0.00007
165	Atranorin	$C_{19}H_{18}O_8$	479-20-9	374.10	0.00005
166	Ethyl benzoylacetate	$C_{11}H_{12}O_3$	94-02-0	192.08	-0.00005
167	Acetophenone	C_8H_8O	98-86-2	120.06	-0.00003
168	Aceglatone	$C_{10}H_{10}O_8$	642-83-1	258.04	0.00003
169	7-Benzyl-2-(4-bromophenyl)-6-methylindolizine	$C_{22}H_{18}BrN$	ChemSpider ID 549718	375.06	0.00129
170	6-Oxododecanedioic acid	$C_{12}H_{20}O_5$	4132-62-1	244.13	0.00003
171	6-Acetyl-4,5,7,8-tetrahydroxynaphthalene-1,2-dione	$C_{12}H_8O_7$	3718-80-7	264.03	-0.00078
172	5-Benzylidene-2,2-dimethyl-1,3-dioxane-4,6-dione	$C_{13}H_{12}O_4$	1214-54-6	232.07	-0.00031
173	Spinochrome M	$C_{12}H_{10}O_6$	2808-46-0	250.05	-0.00007
174	4-Phenolsulfonic acid	$C_6H_6O_4S$	98-67-9	174.00	0.00036
175	Vanillin 4-sulfate	$C_8H_8O_6S$	744984-09-6	232.00	-0.00016
176	4,6-Di-tert-Butylpyrogallol	$C_{14}H_{22}O_3$	3934-77-8	238.16	0.00006
177	3-Decyloxolane-2,5-dione	$C_{14}H_{24}O_3$	18470-76-3	240.17	0.00007
178	3,5-Ditert-butyl-4-hydroxybenzaldehyde	$C_{15}H_{22}O_2$	1620-98-0	234.16	-0.00011

NO	Name	Formula	CAS	Molecular Weight (Da)	Annot.Delta Mass (Da)
179	Schizotenuin F	$C_{28}H_{24}O_{12}$	127498-36-6	552.13	-0.00066
180	2-O-Acetylmalic Anhydride	$C_6H_6O_5$	24766-96-9	158.02	-0.00048
181	2-Methyl-3-(2-pyridin-4-yl-thiazol-4-yl)-1H-indole	$C_{17}H_{13}N_3S$	ChemSpider ID 620846	291.08	-0.00099
182	2-Hydroxy-6-ketononadienedioic acid	$C_9H_{10}O_6$	ChemSpider ID 4444276	214.05	-0.00042
183	2-Acetoxy-3-[3-(4-hydroxy-phenyl)-acryloyloxy]-succinic acid	$C_{15}H_{14}O_9$	106928-35-2	338.06	-0.00042
184	2,7-Dimethoxynaphthalene	$C_{12}H_{12}O_2$	3469-26-9	188.08	-0.00002
185	1-O-Galloylglycerol	$C_{10}H_{12}O_7$	87087-60-3	244.06	-0.00044
186	2,3,4,5-Tetrahydroxy-N-nonylpentanamide	$C_{14}H_{29}NO_5$	ChemSpider ID 480198	291.20	0.00004
187	2-(4-Methoxyphenyl)-1,3-dithiolane	$C_{10}H_{12}OS_2$	6712-20-5	212.03	-0.00050
188	2-(2-(Benzoyloxy)propoxy)propyl benzoate	$C_{20}H_{22}O_5$	20109-39-1	342.15	-0.00046
189	1’-Acetoxychavicol acetate	$C_{13}H_{14}O_4$	52946-22-2	234.09	-0.00031
190	1,4,7,10,13-Pentaoxacyclopentadecane	$C_{10}H_{20}O_5$	33100-27-5	220.13	0.00017
191	15,15’-Bi(1,4,7,10,13-pentaoxacyclohexadecane)	$C_{22}H_{42}O_{10}$	ChemSpider ID 487416	466.28	-0.00029
192	1,6-Hexanediol diacrylate	$C_{12}H_{18}O_4$	13048-33-4	226.12	-0.00023
193	1,2,3,4,5,6-Heptanehexayl hexaacetate	$C_{19}H_{28}O_{12}$	ChemSpider ID 470156	448.16	-0.00036
194	1-(4-Chloro-2-nitrophenyl)piperidine	$C_{11}H_{13}ClN_2O_2$	33784-44-0	240.07	0.00032
195	2-Amino-2-methylpropanenitrile	$C_6H_{11}NO_4$	19355-69-2	161.07	0.00005
196	(Hydroxyethyl)methacrylate	$C_6H_{10}O_3$	868-77-9	130.06	0.00018
197	(2-Dodecen-1-yl)succinic anhydride	$C_{16}H_{26}O_3$	19780-11-1	266.19	0.00005
198	Riboflavin	$C_{17}H_{20}N_4O_6$	83-88-5	376.14	0.00037
199	1,3-Dicaffeoylquinic acid	$C_{25}H_{24}O_{12}$	30964-13-7	516.13	-0.00072
200	Hydroquinone	$C_6H_6O_2$	123-31-9	110.04	-0.00032
201	Eugenol	$C_{10}H_{12}O_2$	97-53-0	164.08	-0.00007
202	Curcumin II	$C_{20}H_{18}O_5$	22608-11-3	338.12	-0.00007
203	Theophylline	$C_7H_8N_4O_2$	58-55-9	180.06	-0.00032
204	Paucin	$C_{23}H_{32}O_{10}$	26836-43-1	468.20	-0.00207
205	Vanilloloside	$C_{14}H_{20}O_8$	74950-96-2	316.12	0.00002
206	Sweroside	$C_{16}H_{22}O_9$	14215-86-2	358.13	-0.00036
207	Sinapaldehyde glucoside	$C_{17}H_{22}O_9$	154461-65-1	370.13	0.00005
208	(2S)-2-(beta-D-Glucopyranosyloxy)-3-methyl-3-butenenitrile	$C_{11}H_{17}NO_6$	66871-89-4	259.11	-0.00023
209	Piceid	$C_{20}H_{22}O_8$	65914-17-2	390.13	0.00024

NO	Name	Formula	CAS	Molecular Weight (Da)	Annot.Delta Mass (Da)
210	Neohesperidose	$C_{12}H_{22}O_{10}$	17074-02-1	326.12	-0.00028
211	Methyl 2,3,6-tri-O-acetyl-4-O-octylhexopyranoside	$C_{21}H_{36}O_9$	ChemSpider ID 470432	432.24	-0.00021
212	Methyl 2,3,4-tri-O-acetyl-1-O-benzoylhexopyranuronate	$C_{20}H_{22}O_{11}$	ChemSpider ID 494836	438.12	-0.00013
213	Khellol glucoside	$C_{19}H_{20}O_{10}$	17226-75-4	408.11	-0.00016
214	Indoxyl beta-D-glucoside	$C_{14}H_{17}NO_6$	487-60-5	295.11	-0.00059
215	alpha-D-Mannose	$C_6H_{12}O_6$	7296-15-3	180.06	0.00008
216	1-Galloyl-beta-D-glucose	$C_{13}H_{16}O_{10}$	13405-60-2	332.07	-0.00030
217	Geranyl beta-D-glucopyranoside	$C_{16}H_{28}O_6$	22850-13-1	316.19	0.00027
218	Eriocitrin	$C_{27}H_{32}O_{15}$	13463-28-0	596.17	-0.00097
219	Dihydrophaseic acid 4-O-beta-D-glucoside	$C_{21}H_{32}O_{10}$	ChemSpider ID 10160748	444.20	-0.00028
220	Caffeic acid 3-glucoside	$C_{15}H_{18}O_9$	24959-81-7	342.09	-0.00041
221	beta-Syringin	$C_{17}H_{24}O_9$	118-34-3	372.14	-0.00017
222	beta-D-Glucose pentaacetate	$C_{16}H_{22}O_{11}$	604-69-3	390.12	-0.00011
223	Benzyl beta-primeveroside	$C_{18}H_{26}O_{10}$	130622-31-0	402.15	-0.00073
224	Apiin	$C_{26}H_{28}O_{14}$	ChemSpider ID 4444321	564.15	-0.00160
225	5,6-Bis-O-(2-oxopropyl)hexose	$C_{12}H_{20}O_8$	ChemSpider ID 468771	292.12	0.00020
226	Dihydrocaffeic acid 3-O-glucuronide	$C_{15}H_{18}O_{10}$	1187945-71-6	358.09	-0.00025
227	Dihydroferulic acid 4-O-glucuronide	$C_{16}H_{20}O_{10}$	86321-28-0	372.11	-0.00038
228	2-Methoxyphenyl 6-O-beta-D-xylopyranosyl-beta-D-glucopyranoside	$C_{18}H_{26}O_{11}$	ChemSpider ID 58837448	418.15	-0.00019
229	2-Hydroxy-2-(4-methyl-2-oxocyclohexyl)propyl hexopyranoside	$C_{16}H_{28}O_8$	ChemSpider ID 30791612	348.18	-0.00006
230	2-Acetyl-1,3,4-tri-O-acetyl-2-deoxypentopyranose	$C_{13}H_{18}O_8$	ChemSpider ID 470300	302.10	-0.00024
231	2,3-Di-O-benzyl-5-O-(4-nitrobenzoyl)pentofuranose	$C_{26}H_{25}NO_8$	ChemSpider ID 488596	479.16	-0.00026
232	2(R)-Hydroxy-2-methylbutyronitrile-beta-D-glucopyranoside	$C_{11}H_{19}NO_6$	534-67-8	261.12	-0.00007
233	1,6-Di-O-galloyl-b-D-glucopyranose	$C_{20}H_{20}O_{14}$	23363-08-8	484.08	-0.00128
234	1,3,4,6-Tetra-O-acetyl-a-D-glucopyranose	$C_{14}H_{20}O_{10}$	13036-15-2	348.11	-0.00042

NO	Name	Formula	CAS	Molecular Weight (Da)	Annot.Delta Mass (Da)
235	2-Deoxy-beta-D-arabino-hexopyranose tetraacetate	$C_{14}H_{20}O_9$	16750-06-4	332.11	-0.00012
236	1,2-Di-O-acetyl-3,5-di-O-benzoylpentofuranose	$C^{23}H^{22}O^{9}$	ChemSpider ID 494887	442.13	-0.00048
237	geniposide	$C_{17}H_{24}O_{10}$	24512-63-8	388.14	-0.00039
238	Tripdiolide	$C_{20}H_{24}O_7$	38647-10-8	376.15	-0.00030
239	Picrotin	$C_{15}H_{18}O_7$	21416-53-5	310.11	0.00016
240	20(S)-Ginsenoside	$C_{36}H_{62}O_8$	78214-33-2	622.44	-0.00131
241	Lamiide	$C_{17}H_{26}O_{12}$	27856-54-8	422.14	-0.00016
242	Gibberellin A7	$C_{19}H_{22}O_5$	510-75-8	330.15	-0.00031
243	Gibberellin A53	$C_{20}H_{28}O_5$	51576-08-0	348.19	0.00000
244	Deoxyloganic acid	$C_{16}H_{24}O_9$	22487-36-1	360.14	-0.00048
245	Brusatol	$C_{26}H_{32}O_{11}$	14907-98-3	520.19	-0.00083
246	Artemotil	$C_{17}H_{28}O_5$	75887-54-6	312.19	-0.00009
247	Allamandin	$C_{15}H_{16}O_7$	51820-82-7	308.09	0.00025
248	(+)-Gibberellic acid	$C_{19}H_{22}O_6$	77-06-5	346.14	0.00016
249	Sebacic acid	$C_{10}H_{18}O_4$	111-20-6	202.12	-0.00002
250	Vanillyl mandelic acid	$C_9H_{10}O_5$	55-10-7	198.05	-0.00022
251	Porphobilinogen	$C_{10}H_{14}N_2O_4$	487-90-1	226.10	0.00010
252	Piscidic acid	$C_{11}H_{12}O_7$	35388-57-9	256.06	0.00002
253	Phthalic acid	$C_8H_6O_4$	88-99-3	166.03	-0.00033
254	Butyl levulinate	$C_9H_{16}O_3$	2052-15-5	172.11	0.00017
255	N-Succinyl-L-diaminopimelic acid	$C_{11}H_{18}N_2O_7$	26605-36-7	290.11	0.00008
256	2-Naphthalen-2-yloxypropanoic acid	$C_{13}H_{12}O_3$	10470-82-3	216.08	0.00009
257	N,N-Diacetyllegionaminic acid	$C_{13}H_{22}N_2O_8$	ChemSpider ID 10179688	334.14	0.00012
258	p-Tolyl beta-D-glucuronide	$C_{13}H_{16}O_7$	17680-99-8	284.09	0.00074
259	Dibenzoyl-D-tartaric acid	$C_{18}H_{14}O_8$	17026-42-5	358.07	-0.00010
260	3,6,9-Trioxaundecanedioic acid	$C_8H_{14}O_7$	13887-98-4	222.07	0.00016
261	Luteic acid	$C_{14}H_8O_9$	476-67-5	320.02	-0.00013
262	Benzyl formate	$C_8H_8O_2$	104-57-4	136.05	0.00006
263	4-[2-(4-Amino-4-carboxybutanoyl)hydrazino]benzoic acid	$C_{12}H_{15}N_3O_5$	69644-85-5	281.10	0.00068
264	4-Hydroxyquinoline-2-carboxylic acid	$C_{10}H_7NO_3$	492-27-3	189.04	-0.00030
265	2-Isopropylmalic acid	$C_7H_{12}O_5$	3237-44-3	176.07	-0.00003
266	Hopantenic acid	$C_{10}H_{19}NO_5$	18679-90-8	233.13	-0.00001
267	Homovanillic acid	$C_9H_{10}O_4$	306-08-1	182.06	0.00029
268	Dimethyl succinylsuccinate	$C_{10}H_{12}O_6$	6289-46-9	228.06	0.00005
269	Glutaric anhydride	$C_5H_6O_3$	108-55-4	114.03	0.00006
270	Glutaric acid	$C_5H_8O_4$	110-94-1	132.04	-0.00009

NO	Name	Formula	CAS	Molecular Weight (Da)	Annot.Delta Mass (Da)
271	Fukiic acid	$C_{11}H_{12}O_8$	35388-56-8	272.05	-0.00039
272	D-Xylonic acid	$C_5H_{10}O_6$	4172-43-4	166.05	0.00029
273	D-Pantothenic acid	$C_9H_{17}NO_5$	79-83-4	219.11	-0.00031
274	DL-Mevalonic acid	$C_6H_{12}O_4$	150-97-0	148.07	-0.00004
275	Di-(2-ethylhexyl)phosphoric acid	$C_{16}H_{35}O_4P$	298-07-7	322.23	0.00003
276	D-Gluconic acid	$C_6H_{12}O_7$	526-95-4	196.06	-0.00029
277	Cinnamic acid	$C_9H_8O_2$	140-10-3	148.05	0.00001
278	Chorismic acid	$C_{10}H_{10}O_6$	617-12-9	226.05	0.00017
279	Brassylic acid	$C_{13}H_{24}O_4$	505-52-2	244.17	-0.00021
280	Benzoic acid	$C_7H_6O_2$	65-85-0	122.04	0.00023
281	Azelaic acid	$C_9H_{16}O_4$	123-99-9	188.10	-0.00004
282	Acetylsalicylic acid	$C_9H_8O_4$	50-78-2	180.04	0.00013
283	5-Methoxy-3-indoleaceate	$C_{11}H_{11}NO_3$	3471-31-6	205.07	-0.00035
284	5-[(3-Methoxyphenoxy)methyl]-2-furoic acid	$C_{13}H_{12}O_5$	405897-57-6	248.07	-0.00043
285	5,5'-Dehydrodivanillate	$C_{16}H_{14}O_8$	2134-90-9	334.07	-0.00027
286	5-(3,4-Dimethoxyphenyl)-2-methyl-3-furoic acid	$C_{14}H_{14}O_5$	696627-31-3	262.08	-0.00022
287	4-oxo-1,2-Cyclopentanedicarboxylic acid	$C_7H_8O_5$	1703-61-3	172.04	0.00025
288	4-Methylsulfonylbenzoic acid	$C_8H_8O_4S$	4052-30-6	200.01	-0.00013
289	4-Carboxy-3-hydroxyphenyl hexopyranosiduronic acid	$C_{13}H_{14}O_{10}$	ChemSpider ID 74886630	330.06	-0.00047
290	3-Butene-1,2,3-tricarboxylic acid	$C_7H_8O_6$	26326-05-6	188.03	0.00030
291	3,8,13,17-Tetramethyl-12-vinyl-2,7,18-Porphinetripropionic acid	$C_{35}H_{36}N_4O_6$	30783-27-8	608.26	-0.00031
292	3,4-Dimethoxyhydrocinnamic acid	$C_{11}H_{14}O_4$	2107-70-2	210.09	0.00001
293	3,4,5-Trimethoxyphenylacetic acid	$C_{11}H_{14}O_5$	951-82-6	226.08	-0.00002
294	3,4,5-Trimethoxyhydrocinnamic acid	$C_{12}H_{16}O_5$	25173-72-2	240.10	-0.00013
295	2-Methylcitric acid	$C_7H_{10}O_7$	6061-96-7	206.04	-0.00007
296	2-Furoic acid	$C_5H_4O_3$	88-14-2	112.02	-0.00015
297	2-Amino-4-pyrimidinecarboxylate	$C_5H_4N_3O_2$	ChemSpider ID 24784670	138.03	0.00058
298	2,5-Dihydroxyterephthalic acid	$C_8H_6O_6$	610-92-4	198.02	-0.00044
299	2,3,4,5-Tetramethoxybenzoic acid	$C_{11}H_{14}O_6$	72023-44-0	242.08	0.00020
300	2,2-Bis(hydroxymethyl)propionic acid	$C_5H_{10}O_4$	4767-03-7	134.06	-0.00020
301	2-(Benzoyloxy)-3-hydroxysuccinic acid	$C_{11}H_{10}O_7$	65621-34-3	254.04	-0.00008
302	12-oxo Phytodienoic Acid	$C_{18}H_{28}O_3$	67204-66-4	292.20	0.00008
303	10-Undecenoic acid	$C_{11}H_{20}O_2$	112-38-9	184.15	-0.00005

NO	Name	Formula	CAS	Molecular Weight (Da)	Annot.Delta Mass (Da)
304	1,3,7-Trimethyluric acid	$C_8H_{10}N_4O_3$	5415-44-1	210.08	0.00019
305	3-Dehydroquinic acid	$C_7H_{10}O_6$	10534-44-8	190.05	0.00011
306	(+)-Quinic acid	$C_7H_{12}O_6$	36413-60-2	192.06	-0.00027
307	trans-Ferulic Acid	$C_{10}H_{10}O_4$	1135-24-6	194.06	-0.00024
308	4-Methoxycinnamic acid	$C_{10}H_{10}O_3$	830-09-1	178.06	-0.00017
309	3,4-Dimethoxycinnamic acid	$C_{11}H_{12}O_4$	2316-26-9	208.07	-0.00017
310	(2,2,6,6-Tetramethyl-4-piperidinyl) acetic acid	$C_{11}H_{21}NO_2$	34635-77-3	199.16	0.00010
311	(+)-Abscisic acid	$C_{15}H_{20}O_4$	7773-56-0	264.14	0.00024
312	Shikimic acid	$C_7H_{10}O_5$	138-59-0	174.05	-0.00025
313	pinellic acid	$C_{18}H_{34}O_5$	97134-11-7	330.24	-0.00039
314	Phaseolic acid	$C_{13}H_{12}O_8$	53755-04-7	296.05	0.00006
315	Phaseic acid	$C_{15}H_{20}O_5$	24394-14-7	280.13	-0.00011
316	Dibutyl sebacate	$C_{18}H_{34}O_4$	109-43-3	314.25	-0.00002
317	1-Acetoxy-2-hydroxy-16-heptadecyn-4-one	$C_{19}H_{32}O_4$	24607-10-1	324.23	0.00002
318	Tuliposide B	$C_{11}H_{18}O_9$	19870-33-8	294.09	-0.00013
319	Suberic acid	$C_8H_{14}O_4$	505-48-6	174.09	0.00006
320	Soyacerebroside I	$C_{40}H_{75}NO_9$	114297-20-0	713.54	-0.00325
321	Ricinelaidic acid	$C_{18}H_{34}O_3$	141-22-0	298.25	0.00013
322	Methyl 2-octynoate	$C_9H_{14}O_2$	111-12-6	154.10	0.00001
323	10-Hydroxy-2-decenoic acid	C_{10}H18O3	14113-05-4	186.13	-0.00002
324	Phloionolic acid	$C_{18}H_{36}O_5$	496-86-6	332.26	-0.00014
325	N-Oleoyl-L-Serine	$C_{21}H_{39}NO_4$	1246302-99-7	369.29	0.00007
326	3-Hydroxydecanoic acid	$C_{10}H_{20}O_3$	14292-26-3	188.14	0.00023
327	1-alpha-Linolenoylglycerol	$C_{21}H_{36}O_4$	18465-99-1	352.26	0.00031
328	2-Hydroxydocosanoic acid	$C_{22}H_{44}O_3$	13980-14-8	356.33	0.00062
329	Linolenelaidic acid	$C_{18}H_{30}O_2$	28290-79-1	278.22	0.00000
330	16-Hydroxyhexadecanoic acid	$C_{16}H_{32}O_3$	506-13-8	272.24	0.00008
331	Ethyl 3-oxohexanoate	$C_8H_{14}O_3$	3249-68-1	158.09	-0.00024
332	Dodecanedioic acid	$C_{12}H_{22}O_4$	693-23-2	230.15	-0.00009
333	Diethyl diallylmalonate	$C_{13}H_{20}O_4$	3195-24-2	240.14	0.00008
334	9-Hydroxynonanoic acid	$C_9H_{18}O_3$	3788-56-5	174.13	0.00006
335	9,10-Dihydroxystearic acid	$C_{18}H_{36}O_4$	120-87-6	316.26	0.00008
336	2-Hydroxytetracosanoic acid	$C_{24}H_{48}O_3$	544-57-0	384.36	0.00048
337	13-Hydroxyoctadecadienoic acid	$C_{18}H_{32}O_3$	29623-28-7	296.24	-0.00007
338	13-Hydroxy-9-methoxy-10-oxo-11-octadecenoic acid	$C_{19}H_{34}O_5$	150147-08-3	342.24	-0.00028
339	13(S)-Hydroperoxylinolenic acid	$C_{18}H_{30}O_4$	67597-26-6	310.21	-0.00013
340	12-Hydroxyjasmonic acid glucoside	$C_{18}H_{28}O_9$	120399-24-8	388.17	-0.00016
341	12-Hydroxylauric acid	$C_{12}H_{24}O_3$	505-95-3	216.17	0.00020
342	12-Hydroxyoctadecanoic acid	$C_{18}H_{36}O_3$	106-14-9	300.27	0.00035

NO	Name	Formula	CAS	Molecular Weight (Da)	Annot.Delta Mass (Da)
343	12-Aminolauric Acid	$C_{12}H_{25}NO_2$	693-57-2	215.19	0.00017
344	(5Z,8Z)-Tetradecadienoylcarnitine	$C_{21}H_{37}NO_4$	1469901-01-6	367.27	0.00006
345	LPE(18:3)	$C_{23}H_{42}NO_7P$	ChemSpider ID 24769359	475.27	0.00011
346	(10E,15Z)-9,12,13-Trihydroxy-10,15-octadecadienoic acid	$C_{18}H_{32}O_5$	95341-44-9	328.22	-0.00040
347	Pentadecanedioic acid	$C_{15}H_{28}O_4$	1460-18-0	272.20	0.00011
348	N-Undecanoylglycine	$C_{13}H_{25}NO_3$	83871-09-4	243.18	-0.00069
349	N-Lauroylglycine	$C_{14}H_{27}NO_3$	7596-88-5	257.20	-0.00014

Note: Annot. Delta Mass represents the difference between the actual measured value of molecular weight and the theoretical value.

Table S3. List of 206 non-volatile metabolites in the processing of Rizhao green tea (ESI+, Hilic column).

NO	Name	Formula	CAS	Molecular Weight (Da)	Annot.Delta Mass (Da)
1	N^2-Acetyl-N-methyllysinamide	$C_9H_{19}N_3O_2$	ChemSpider ID 485364	201.15	-0.00033
2	N^2-(tert-Butoxycarbonyl)glutamin	$C_{10}H_{18}N_2O_5$	85535-45-1	246.12	-0.00041
3	N-Acetylvaline	$C_7H_{13}NO_3$	3067-19-4	159.09	-0.00013
4	N-Acetyl-L-leucine	$C_8H_{15}NO_3$	1188-21-2	173.11	-0.00012
5	N-Acetyl-L-aspartic acid	$C_6H_9NO_5$	997-55-7	175.05	-0.00020
6	L-alpha-Aspartyl-D-phenylalanine methyl ester	$C_{14}H_{18}N_2O_5$	22839-65-2	294.12	-0.00029
7	Leu-Pro	$C_{11}H_{20}N_2O_3$	6403-35-6	228.15	-0.00030
8	L-Theanine	$C_7H_{14}N_2O_3$	3081-61-6	174.10	-0.00018
9	L-Pyroglutamic acid	$C_5H_7NO_3$	98-79-3	129.04	-0.00006
10	L-Proline	$C_5H_9NO_2$	147-85-3	115.06	-0.00020
11	L-Valine	$C_5H_{11}NO_2$	72-18-4	117.08	-0.00032
12	L-Leucine	$C_6H_{13}NO_2$	61-90-5	131.09	-0.00029
13	Homocycloleucine	$C_7H_{13}NO_2$	2756-85-6	143.09	-0.00027
14	Gly-Leu	$C_8H_{16}N_2O_3$	869-19-2	188.12	-0.00030
15	Ethyl DL-methionate	$C_7H_{15}NO_2S$	452-95-9	177.08	-0.00020
16	Boc-Glycine	$C_7H_{13}NO_4$	4530-20-5	175.08	-0.00005
17	Acetyl-L-proline	$C_7H_{11}NO_3$	68-95-1	157.07	-0.00018
18	5-Hydroxy-DL-tryptophan	$C_{11}H_{12}N_2O_3$	56-69-9	220.08	-0.00042
19	2-Morpholinoacetic acid	$C_6H_{11}NO_3$	3235-69-6	145.07	-0.00027
20	N-tert-Butoxycarbonyl-DL-proline	$C_{10}H_{17}NO_4$	59433-50-0	215.12	-0.00021
21	N-Ethoxycarbonyl-L-proline	$C_8H_{13}NO_4$	5700-74-3	187.08	0.00018
22	Hydroginkgolic acid	$C_{22}H_{36}O_3$	16611-84-0	348.27	-0.00068
23	Uracil	$C_4H_4N_2O_2$	66-22-8	112.03	-0.00023
24	trans-Zeatin	$C_{10}H_{13}N_5O$	1637-39-4	219.11	-0.00025
25	4-Amino-5-hydroxymethyl-2-methy lpyrimidine	$C_6H_9N_3O$	73-67-6	139.07	-0.00018
26	Thymine	$C_5H_6N_2O_2$	65-71-4	126.04	-0.00019
27	Xanthine	$C_5H_4N_4O_2$	69-89-6	152.03	-0.00010
28	Purine	$C_5H_4N_4$	120-73-0	120.04	-0.00018
29	N6-Methyladenosine	$C_{11}H_{15}N_5O_4$	1867-73-8	281.11	-0.00061
30	N,N-Dimethyladenine	$C_7H_9N_5$	938-55-6	163.09	-0.00014
31	1-Methylxanthine	$C_6H_6N_4O_2$	6136-37-4	166.05	-0.00028
32	Hypoxanthin	$C_5H_4N_4O$	68-94-0	136.04	-0.00027
33	Cytosine	$C_4H_5N_3O$	71-30-7	111.04	-0.00021
34	5'-Deoxy-5'-methylthioadenosine	$C_{11}H_{15}N_5O_3S$	2457-80-9	297.09	-0.00050
35	Adenine	$C_5H_5N_5$	73-24-5	135.05	-0.00031
36	9-(2-Hydroxypropyl)adenine	$C_8H_{11}N_5O$	14047-26-8	193.10	-0.00021

NO	Name	Formula	CAS	Molecular Weight (Da)	Annot.Delta Mass (Da)
37	8-Hydroxyguanine	$C_5H_5N_5O_2$	5614-64-2	167.04	-0.00016
38	6-O-Methylguanine	$C_6H_7N_5O$	20535-83-5	165.06	-0.00012
39	5-Methylcytosine	$C_5H_7N_3O$	554-01-8	125.06	-0.00016
40	3-Methyladenine	$C_6H_7N_5$	5142-23-4	149.07	-0.00017
41	N9-Hydroxyethyladenine	$C_7H_9N_5O$	707-99-3	179.08	-0.00032
42	2’-Deoxyadenosine	$C_{10}H_{13}N_5O_3$	958-09-8	251.10	-0.00040
43	3,4,5,7,3’,4’,5’-Heptahydroxyflavan	$C_{15}H_{14}O_8$	491-52-1	322.07	-0.00048
44	Quercitrin	$C_{21}H_{20}O_{11}$	522-12-3	448.10	-0.00081
45	Kaempferol	$C_{15}H_{10}O_6$	520-18-3	286.05	-0.00063
46	3’,4’,5,7-Tetrahydroxyflavanone	$C_{15}H_{12}O_6$	4049-38-1	288.06	-0.00063
47	(+)-Catechin	$C_{15}H_{14}O_6$	154-23-4	290.08	-0.00062
48	(+)-Procyanidin B2	$C_{30}H_{26}O_{12}$	29106-49-8	578.14	-0.00053
49	N,N-Diethyl-4-nitroaniline	$C_{10}H_{14}N_2O_2$	2216-15-1	194.11	-0.00009
50	Vanillin	$C_8H_8O_3$	121-33-5	152.05	-0.00016
51	L-Valinol	$C_5H_{13}NO$	2026-48-4	103.10	-0.00029
52	Methyl propiolate	$C_4H_4O_2$	922-67-8	84.02	-0.00019
53	Trimethylamine N-oxide	C_3H_9NO	1184-78-7	75.07	-0.00008
54	2,2,6,6-Tetramethyl-4-piperidinone	$C_9H_{17}NO$	826-36-8	155.13	-0.00013
55	1-Methylpiperazine	$C_5H_{12}N_2$	109-01-3	100.10	-0.00012
56	Ethyl piperazine-1-carboxylate	$C_7H_{14}N_2O_2$	120-43-4	158.11	-0.00012
57	Thiodiglycol	$C_4H_{10}O_2S$	111-48-8	122.04	-0.00019
58	Tetralin	$C_{10}H_{12}$	119-64-2	132.09	-0.00016
59	Tetraethylene glycol dimethyl ether	$C_{10}H_{22}O_5$	143-24-8	222.15	-0.00026
60	N-Boc-N-methyl-aminoethanol	$C_8H_{17}NO_3$	57561-39-4	175.12	-0.00019
61	4-Methyl-5-thiazoleethanol	C_6H_9NOS	137-00-8	143.04	-0.00014
62	Succinimide	$C_4H_5NO_2$	123-56-8	99.03	-0.00015
63	4-Acetoxystyrene	$C_{10}H_{10}O_2$	2628-16-2	162.07	-0.00030
64	Pentaethylene glycol	$C_{10}H_{22}O_6$	4792-15-8	238.14	-0.00039
65	Tetraethylene glycol	$C_8H_{18}O_5$	112-60-7	194.12	-0.00024
66	p-Anisoin	$C_{16}H_{16}O_4$	119-52-8	272.10	-0.00059
67	Octylamine	$C_8H_{19}N$	111-86-4	129.15	-0.00012
68	Octinoxate	$C_{18}H_{26}O_3$	5466-77-3	290.19	-0.00076
69	3-Acetylindole	$C_{10}H_9NO$	703-80-0	159.07	-0.00016
70	N-Nitropyrrolidine	$C_4H_8N_2O$	930-55-2	100.06	-0.00019
71	N-Isopropylacrylamide	$C_6H_{11}NO$	2210-25-5	113.08	-0.00029
72	Decylamine	$C_{10}H_{23}N$	2016-57-1	157.18	-0.00005
73	N-Methylhomoveratrylamine	$C_{11}H_{17}NO_2$	3490-06-0	195.13	-0.00012
74	N,N-Dimethylacetamide	C_4H_9NO	127-19-5	87.07	-0.00011
75	N,N-Dibutylethanolamine	$C_{10}H_{23}NO$	102-81-8	173.18	-0.00017
76	N-Lauryldiethanolamine	$C_{16}H_{35}NO_2$	1541-67-9	273.27	-0.00061
77	Methyl ricinoleate	$C_{19}H_{36}O_3$	141-24-2	312.27	-0.00063
78	Methyl	$C_{21}H_{43}NO_2$	56817-90-4	341.33	-0.00062

NO	Name	Formula	CAS	Molecular Weight (Da)	Annot.Delta Mass (Da)
	6-(dimethylamino)octadecanoate				
79	Piperidine-2,6-dione	$C_5H_7NO_2$	1121-89-7	113.05	-0.00010
80	Laurylamine	$C_{12}H_{27}N$	124-22-1	185.21	-0.00018
81	Laurophenone	$C_{18}H_{28}O$	1674-38-0	260.21	-0.00038
82	Lauramide	$C_{12}H_{25}NO$	1120-16-7	199.19	-0.00031
83	4-Piperidinecarboxamide	$C_6H_{12}N_2O$	39546-32-2	128.09	-0.00019
84	Indole	C_8H_7N	120-72-9	117.06	-0.00021
85	Heptaethylene Glycol	$C_{14}H_{30}O_8$	5617-32-3	326.19	-0.00052
86	1-Methylcyclohexanol	$C_8H_{12}O$	590-67-0	124.09	-0.00019
87	2-Hydroxyquinoline	C_9H_7NO	59-31-4	145.05	-0.00015
88	Ethyl 4-hydroxy-2-methylpyrimidine-5-carboxylate	$C_8H_{10}N_2O_3$	53135-24-3	182.07	-0.00046
89	Ethyl 2-oxocyclohexanecarboxylate	$C_9H_{14}O_3$	1655-07-8	170.09	-0.00031
90	Ethyl 2-(hydroxyimino)-3-oxobutanoate	$C_6H_9NO_4$	5408-04-8	159.05	-0.00018
91	2,6-Ditert-butyl-4-(hydroxymethyl) phenol	$C_{15}H_{24}O_2$	88-26-6	236.18	-0.00043
92	N,N-Dimethylformamide	C_3H_7NO	68-12-2	73.05	-0.00011
93	Dimethyl N-acetyl-L-aspartate	$C_8H_{13}NO_5$	57289-64-2	203.08	-0.00015
94	Dimethyl 2-methylenesuccinate	$C_7H_{10}O_4$	617-52-7	158.06	-0.00012
95	Diisopropyl sulfite	$C_6H_{14}O_3S$	4773-13-1	166.07	-0.00009
96	Diethyl 4-oxo-4H-quinolizine-1,3-dicarboxylate	$C_{15}H_{15}NO_5$	54401-76-2	289.09	-0.00068
97	Dielaidoylphosphatidylcholine	$C_{44}H_{84}NO_8P$	52088-89-8	785.59	-0.00342
98	Choline O-Sulfate	$C_5H_{13}NO_4S$	4858-96-2	183.06	-0.00031
99	Caprylic diethanolamide	$C_{12}H_{25}NO_3$	3077-30-3	231.18	-0.00038
100	Butyl Sulfite	$C_8H_{18}O_3S$	626-85-7	194.10	-0.00056
101	Butyl cyclohexyl phthalate	$C_{18}H_{24}O_4$	84-64-0	304.17	-0.00064
102	Butoxytriglycol	$C_{10}H_{22}O_4$	143-22-6	206.15	-0.00018
103	Ethyl benzoylacetate	$C_{11}H_{12}O_3$	94-02-0	192.08	-0.00021
104	Acetophenone	C_8H_8O	98-86-2	120.06	-0.00023
105	4-Heptyloxyphenol	$C_{13}H_{20}O_2$	13037-86-0	208.15	-0.00030
106	6-Hydroxymethyl-7,8-dihydropterin	$C_7H_9N_5O_2$	3672-03-5	195.08	-0.00003
107	6,7-Dimethylpterin	$C_8H_9N_5O$	611-55-2	191.08	-0.00020
108	5-Hydroxyindole	C_8H_7NO	1953-54-4	133.05	-0.00004
109	5-(4-Methoxyphenyl)-1,3-cyclohexanedione	$C_{13}H_{14}O_3$	1774-12-5	218.09	-0.00054
110	4-Vinylpyridine	C_7H_7N	100-43-6	105.06	-0.00016
111	4-Nitroaniline	$C_6H_6N_2O_2$	100-01-6	138.04	-0.00017
112	p-Anisidine	C_7H_9NO	104-94-9	123.07	0.00008

NO	Name	Formula	CAS	Molecular Weight (Da)	Annot.Delta Mass (Da)
113	4-Aminophenol	C_6H_7NO	123-30-8	109.05	-0.00014
114	4,6-Di-tert-butylpyrogallol	$C_{14}H_{22}O_3$	3934-77-8	238.16	-0.00043
115	3-Methyl-5-pyrazolone	$C_4H_6N_2O$	108-26-9	98.05	-0.00013
116	5-Methyl-3,4-dihydropyrazole-2-carboxamide	$C_5H_9N_3O$	17014-30-1	127.07	-0.00023
117	3-Ethyl-o-xylene	$C_{10}H_{14}$	933-98-2	134.11	-0.00022
118	2-tert-Butyl-4-methoxyphenol	$C_{11}H_{16}O_2$	121-00-6	180.11	-0.00025
119	3-Amino-benzamide	$C_7H_8N_2O$	3544-24-9	136.06	-0.00011
120	3-Hydroxyundecanoyl carnitine	$C_{18}H_{35}NO_5$	ChemSpider ID 59664326	345.25	-0.00071
121	3,5-Ditert-butyl-4-hydroxybenzonitrile	$C_{15}H_{21}NO$	1988-88-1	231.16	-0.00048
122	3,5-Ditert-butyl-4-hydroxybenzaldehyde	$C_{15}H_{22}O_2$	1620-98-0	234.16	-0.00053
123	3,5-Dimethoxybenzohydrazide	$C_9H_{12}N_2O_3$	51707-38-1	196.08	-0.00060
124	3,5-Ditert-butyl-4-hydroxyacetophenone	$C_{16}H_{24}O_2$	14035-33-7	248.18	-0.00038
125	2-Pyrrolidone	C_4H_7NO	616-45-5	85.05	-0.00014
126	2-Morpholinoacetonitrile	$C_6H_{10}N_2O$	5807-02-3	126.08	-0.00019
127	2-Methoxy-5-nitrophenol	$C_7H_7NO_4$	636-93-1	169.04	-0.00023
128	2-Amino-1-(piperidin-1-yl)propan-1-one	$C_8H_{16}N_2O$	805974-19-0	156.13	-0.00013
129	2-Amino-1,3,4-octadecanetriol	$C_{18}H_{39}NO_3$	13552-11-9	317.29	-0.00052
130	N-Methylmoranoline	$C_7H_{15}NO_4$	69567-10-8	177.10	-0.00010
131	2,4,6-Triaminotoluene	$C_7H_{11}N_3$	88-02-8	137.10	-0.00013
132	1-Vinyl-2-pyrrolidone	C_6H_9NO	88-12-0	111.07	-0.00023
133	1-Methyl-4-nitrosopiperazine	$C_5H_{11}N_3O$	16339-07-4	129.09	-0.00017
134	4-Amino-1-carbethoxypiperidine	$C_8H_{16}N_2O_2$	58859-46-4	172.12	-0.00019
135	1-Boc-piperazine	$C_9H_{18}N_2O_2$	57260-71-6	186.14	-0.00030
136	1-Boc-3-piperidinol	$C_{10}H_{19}NO_3$	85275-45-2	201.14	-0.00026
137	1,8,15,22-Tetraazacyclooctacosane-2,9,16,23-tetrone	$C_{24}H_{44}N_4O_4$	5834-63-9	452.34	-0.00103
138	1,6-Hexanediol diacrylate	$C_{12}H_{18}O4$	13048-33-4	226.12	-0.00048
139	1,3,5-Norcaratriene	C_7H_6	4646-69-9	90.05	-0.00017
140	1,1-Bis(morpholino)ethylene	$C_{10}H_{18}N_2O_2$	14212-87-4	198.14	-0.00020
141	Diisopropyl diazene-1,2-dicarboxylate	$C_8H_{14}N_2O_4$	2446-83-5	202.10	-0.00015
142	Yomogiartemin	$C_{17}H_{20}O_7$	69684-72-6	336.12	-0.00059
143	Pyridoxal	$C_8H_9NO_3$	66-72-8	167.06	-0.00021
144	(+)-Riboflavin	$C_{17}H_{20}N_4O_6$	83-88-5	376.14	-0.00072
145	Niacin	$C_6H_5NO_2$	59-67-6	123.03	-0.00008
146	Nicotinamide	$C_6H_6N_2O$	98-92-0	122.05	-0.00024

NO	Name	Formula	CAS	Molecular Weight (Da)	Annot.Delta Mass (Da)
147	Dopamine	$C_8H_{11}NO_2$	51-61-6	153.08	-0.00019
148	Theophylline	$C_7H_8N_4O_2$	58-55-9	180.06	-0.00034
149	Terminaline	$C_{23}H_{41}NO_2$	15112-49-9	363.31	-0.00080
150	4-Butyrobetaine	$C_7H_{15}NO_2$	ChemSpider ID 705	145.11	-0.00025
151	Oxyphylline	$C_9H_{12}N_4O_3$	519-37-9	224.09	-0.00027
152	Caffeine	$C_8H_{10}N_4O_2$	58-08-2	194.08	-0.00034
153	Arecoline	$C_8H_{13}NO_2$	63-75-2	155.09	-0.00015
154	Sorbic acid	$C_6H_8O_2$	110-44-1	112.05	-0.00018
155	Quinolinic acid	$C_7H_5NO_4$	89-00-9	167.02	-0.00014
156	Pheophorbide A	$C_{35}H_{36}N_4O_5$	15664-29-6	592.27	-0.00134
157	4-Hexyloxybenzoic acid	$C_{13}H_{18}O_3$	1142-39-8	222.13	-0.00040
158	Oxamide	$C_2H_4N_2O_2$	471-46-5	88.03	-0.00011
159	Indole-3-acetic acid	$C_{10}H_9NO_2$	87-51-4	175.06	-0.00019
160	gamma-Aminobutyric acid	$C_4H_9NO_2$	56-12-2	103.06	-0.00015
161	Boc-GABA	$C_9H_{17}NO_4$	57294-38-9	203.12	-0.00023
162	Guvacine	$C_6H_9NO_2$	498-96-4	127.06	-0.00025
163	Gibberellin A7	$C_{19}H_{22}O_5$	510-75-8	330.15	-0.00068
164	Alantolactone	$C_{15}H_{20}O_2$	546-43-0	232.15	-0.00054
165	Coumarin	$C_9H_6O_2$	91-64-5	146.04	-0.00016
166	Butyl levulinate	$C_9H_{16}O_3$	2052-15-5	172.11	-0.00023
167	Benzyl formate	$C_8H_8O_2$	104-57-4	136.05	-0.00017
168	D-Pantothenic acid	$C_9H_{17}NO_5$	79-83-4	219.11	-0.00024
169	Chrysanthemic acid	$C_{10}H_{16}O_2$	10453-89-1	168.11	-0.00027
170	Aminolevulinic acid	$C_5H_9NO_3$	106-60-5	131.06	-0.00008
171	1-Aminocyclopropanecarboxylic acid	$C_4H_7NO_2$	22059-21-8	101.05	-0.00017
172	4-Aminobenzoic acid	$C_7H_7NO_2$	150-13-0	137.05	-0.00012
173	3-Methylglutarylcarnitine	$C_{13}H_{23}NO_6$	102673-95-0	289.15	0.00079
174	Dibutyl sebacate	$C_{18}H_{34}O_4$	109-43-3	314.25	-0.00036
175	1-Acetoxy-2-hydroxy-16-heptadecyn-4-one	$C_{19}H_{32}O_4$	24607-10-1	324.23	-0.00063
176	(E)-12-Oxo-10-dodecanoic acid	$C_{12}H_{20}O_3$	65410-38-0	212.14	-0.00037
177	Tetrahydrodeoxycorticosterone	$C_{21}H_{34}O_3$	567-02-2	334.25	-0.00071
178	Soyacerebroside I	$C_{40}H_{75}NO_9$	114297-20-0	713.54	-0.00154
179	Ricinelaidic acid	$C_{18}H_{34}O_3$	540-12-5	298.25	-0.00050
180	Methyl 2-octynoate	$C_9H_{14}O_2$	111-12-6	154.10	-0.00042
181	1-alpha-Linolenoyl-2-linoleoyl-phosphatidylcholine	$C_{44}H_{78}NO_8P$	8002-43-5	779.54	-0.00153
182	L-alpha-1-Palmitoyl-2-linoleoylphosphatidylcholine	$C_{42}H_{80}NO_8P$	17708-90-6	757.56	-0.00076
183	Palmitoyl alanine	$C_{19}H_{37}NO_3$	56255-31-3	327.28	-0.00093

NO	Name	Formula	CAS	Molecular Weight (Da)	Annot.Delta Mass (Da)
184	1-alpha-Linolenoylglycerol	$C_{21}H_{36}O_4$	18465-99-1	352.26	-0.00072
185	Linolenelaidic acid	$C_{18}H_{30}O_2$	28290-79-1	278.22	-0.00048
186	L-alpha-Palmitin	$C_{19}H_{38}O_4$	32899-41-5	330.28	-0.00083
187	Embelin	$C_{17}H_{26}O_4$	550-24-3	294.18	-0.00043
188	10-Nitrooleic acid	$C_{18}H_{33}NO_4$	88127-53-1	327.24	-0.00074
189	Biacetyl	$C_4H_6O_2$	431-03-8	86.04	-0.00010
190	Adipamide	$C_6H_{12}N_2O_2$	628-94-4	144.09	-0.00013
191	A-12(13)-EpODE	$C_{18}H_{30}O_3$	ChemSpider ID 17220744	294.22	-0.00065
192	2-Hexenoylcarnitine	$C_{13}H_{23}NO_4$	99452-58-1	257.16	-0.00054
193	13-Hydroxyoctadecadienoic acid	$C_{18}H_{32}O_3$	29623-28-7	296.23	-0.00068
194	13(S)-Hydroperoxylinoleic acid	$C_{18}H_{32}O_4$	33964-75-9	312.23	-0.00075
195	13(S)-HpOTrE;13-Hydroperoxy-9,11E,15Z-octadecatrienoic acid	$C_{18}H_{30}O_4$	ChemSpider ID 4445993	310.21	-0.00072
196	12-Oxo phytodienoic acid	$C_{18}H_{28}O_3$	85551-10-6	292.20	-0.00066
197	12-Aminolauric Acid	$C_{12}H_{25}NO_2$	693-57-2	215.19	-0.00026
198	10-Gingerol	$C_{21}H_{34}O_4$	23513-15-7	350.25	-0.00059
199	PC(16:0/18:3(6Z,9Z,12Z))	$C_{42}H_{78}NO_8P$	203393-39-9	755.55	-0.00039
200	PC(18:1(9Z)/18:2(9Z,12Z))	$C_{44}H_{82}NO_8P$	17041-44-0	783.57	-0.00381
201	PE(18:2(9Z,12Z)/0:0)	$C_{23}H_{44}NO_7P$	85046-18-0	477.28	-0.00079
202	PE(16:0/20:4)	$C_{41}H_{74}NO_8P$	70812-59-8	739.51	-0.00149
203	PE(18:2(9Z,12Z)/18:3(9Z,12Z,15Z))	$C_{41}H_{72}NO_8P$	ChemSpider ID 24768558	737.50	-0.00123
204	(10E,15Z)-9,12,13-Trihydroxy-10,15-octadecadienoic acid	$C_{18}H_{32}O_5$	95341-44-9	328.22	-0.00060
205	N-Undecanoylglycine	$C_{13}H_{25}NO_3$	83871-09-4	243.18	-0.00025
206	7-epi-JasmonicAcid	$C_{12}H_{18}O_3$	62653-85-4	210.13	-0.00028

Note: Annot. Delta Mass represents the difference between the actual measured value of molecular weight and the theoretical value.

Table S4-1. Load matrix of principal components (C18 column, ESI+).

Name	Principal component	
	1	2
Pipecolic acid	9.151	-0.521
L-Norleucine	2.297	2.304
N,N-Diethylglycine	0.688	-0.821
Allysine	-0.257	-0.641
Ethyl methioninate	-0.336	0.705
L-alpha-Glutamyl-L-hydroxyproline	-0.454	0.246
Cianidanol	-0.224	1.095
Quercetin	-0.660	0.315
Myricetin	-0.822	-0.198
6-Methoxyquercetin	-0.762	0.038
ECG	-0.316	2.546
EGCG	-0.237	3.102
3,4-Dihydroxybenzaldehyde	-0.201	2.642
Protocatechuic acid	-0.467	-0.637
3,4-Dihydroxyphenylacetic acid	-0.725	0.185
Gallic acid	-0.779	-0.035
Phloretin	-0.448	-0.417
12-OPDA	-0.622	-0.616
9-KODE	-0.700	-0.598
9-Oxo-ODE	-0.785	-0.594
9-J1-Phytoprostane	-0.836	-0.590
Linoleoyl ethanolamide	-0.646	-0.612
PC(18:3(9Z,12Z,15Z)/18:3(9Z,12Z,15Z))	-0.550	-0.488
Guanine	3.365	-0.315
1-Methylxanthine	-0.429	-0.591
2’-Deoxyadenosine	-0.500	-0.601
Guanosine	1.858	-0.384
5’-S-Methyl-5’-thioadenosine	-0.388	0.537
(2S)-2-Amino-4-hexynoic acid	-0.433	-0.624
(E)-8-Hydroxy-2-octene-4,6-diynoic acid	-0.806	-0.113
2-Hydroxy-6-oxonona-2,4-diene-1,9-dioate	-0.745	-0.561
N-Succinyl-L,L-2,6-diaminopimelate	-0.731	-0.422
Luteic acid	-0.773	0.075
Stachydrine	0.443	-0.729
Methyl gallate	-0.666	0.069
Ellagic acid	-0.828	-0.334
Procyanidin B2	-0.830	-0.348
4-Hydroxycoumarin	-0.464	-0.652
1-Vinyl-2-pyrrolidone	0.568	-0.744
N-Acetylpyrrolidine	-0.317	-0.665
4-Hydroxybenzaldehyde	-0.397	2.073

Name	Principal component	
	1	2
2-Hydroxy-1,4-benzoquinone	-0.509	-0.447
5-Formylsalicylaldehyde	-0.747	0.123
3,4-Dihydroxymandelaldehyde	-0.785	-0.551
Allyl salicylate	-0.780	-0.569
Rhamnopterin	1.181	0.105
Di-2-pentanyl malate	-0.585	-0.513
Pheophorbide A	2.988	-0.230
Initial eigenvalue	2.770	0.946
Variance contribution rate (%)	69.243	23.650

Table S4-2. Load matrix of principal components (C18 column, ESI−).

Name	Principal component	
	1	2
L-Theanine	10.433	-1.369
Methyl gallate	0.839	1.193
Ellagic acid	0.114	0.473
Gallic acid	-0.724	0.039
Digallic acid	-0.585	-0.206
(-)-Epigallocatechin gallate	-0.142	1.215
(-)-Epiafzelechin 3-O-gallate	-0.709	0.084
(-)-Epicatechin gallate	0.337	2.069
Salicylic acid	0.831	1.186
Protocatechuic acid	0.346	-0.885
3,4-Dihydroxyphenylacetic acid	-0.677	-0.154
Theaflavic acid	-0.714	-0.131
4-Hydroxycinnamic acid	0.647	-0.522
Theogallin	-0.083	0.677
3’,4’,5,7-Tetrahydroxyflavanone	-0.651	-0.088
Cianidanol	4.212	1.209
2-(3,4-Dihydroxyphenyl)-5,7-dihydroxy-3,6,8-trimethoxy-4H-chromen-4-one	-0.736	0.027
3,4,5,7,3’,4’,5’-Heptahydroxyflavan	0.472	-0.737
Phloretin	-0.577	-0.188
Gossypin	-0.732	0.039
Dihydromyricetin	-0.708	-0.112
4-Methyl-6,7-diacetoxycoumarin	-0.462	-0.265
Trimethyl 4-hydroxy-5-methyl-1,2,3-benzenetricarboxylate	-0.001	-0.229
Lauric anhydride	-0.687	-0.069
Bis(2-butoxyethyl) adipate	-0.114	-0.024
6-Acetyl-4,5,7,8-tetrahydroxynaphthalene-1,2-dione	0.114	-0.825
2-Hydroxy-6-ketononadienedioic acid	-0.527	-0.220
2-Acetoxy-3-[3-(4-hydroxy-phenyl)-acryloyloxy]-succinic acid	-0.734	-0.108
’-Acetoxychavicol acetate	-0.680	-0.146
1,2,3,4,5,6-Heptanehexayl hexaacetate	-0.710	-0.034
Hydroquinone	-0.481	-0.278
Benzyl beta-primeveroside	-0.689	-0.023
Apiin	-0.014	-0.240
1,6-Di-O-galloyl-b-D-glucopyranose	-0.552	0.372
20(S)-Ginsenoside	-0.578	-0.157
Fukiic acid	-0.629	-0.120
5,5’-Dehydrodivanillate	-0.669	-0.077
2-Amino-4-pyrimidinecarboxylate	0.399	-0.407
2,5-Dihydroxyterephthalic acid	-0.035	-0.432

Name	Principal component	
	1	2
12-oxo Phytodienoic Acid	-0.461	-0.058
pinellic acid	-0.371	-0.058
Phaseolic acid	-0.732	-0.115
16-Hydroxyhexadecanoic acid	-0.523	-0.042
13-Hydroxyoctadecadienoic acid	-0.510	-0.070
13(S)-Hydroperoxylinolenic acid	-0.335	-0.082
12-Hydroxyoctadecanoic acid	-0.718	-0.071
(10E,15Z)-9,12,13-Trihydroxy-10,15-octadecadienoic acid	-0.497	-0.040
Initial eigenvalue	3.083	0.716
Variance contribution rate (%)	77.069	17.907

Table S4-3. Load matrix of principal components (Hilic column, ESI+).

Name	Principal component	
	1	2
L-Leucine	1.739	-0.540
Homocycloleucine	-0.740	0.034
Acetyl-L-proline	-0.796	0.041
2-Morpholinoacetic acid	-0.701	0.033
N6-Methyladenosine	-0.750	0.044
1-Methylxanthine	-0.784	0.043
Hypoxanthin	0.562	-0.260
Cytosine	-0.496	-0.022
Adenine	6.427	-2.477
Thiodiglycol	-0.764	0.046
Octinoxate	-0.666	0.036
N-Isopropylacrylamide	0.225	-0.196
Laurophenone	-0.804	0.050
Choline O-Sulfate	1.469	-0.689
1-Vinyl-2-pyrrolidone	-0.694	0.057
1,3,5-Norcaratriene	5.186	3.353
Niacin	-0.705	0.026
Nicotinamide	-0.559	0.103
Theophylline	-0.495	0.005
4-Butyrobetaine	-0.783	0.048
Pheophorbide A	-0.503	-0.097
Boc-GABA	-0.787	0.048
Guvacine	-0.702	0.033
1-Aminocyclopropanecarboxylic acid	-0.772	0.166
1-alpha-Linolenoylglycerol	-0.736	0.040
Embelin	-0.762	0.029
A-12(13)-EpODE	-0.339	-0.007
13(S)-HpOTrE	-0.796	0.049
12-Oxo phytodienoic acid	-0.475	0.002
Initial eigenvalue	3.108	0.767
Variance contribution rate (%)	77.694	19.168
